# Synthesis and Biological Evaluation of New Pyridothienopyrimidine Derivatives as Antibacterial Agents and *Escherichia coli* Topoisomerase II Inhibitors

**DOI:** 10.3390/antibiotics9100695

**Published:** 2020-10-14

**Authors:** Eman M. Mohi El-Deen, Eman A. Abd El-Meguid, Eman A. Karam, Eman S. Nossier, Marwa F. Ahmed

**Affiliations:** 1 Department of Therapeutic Chemistry, National Research Centre, Dokki, Cairo 12622, Egypt; 2 Department of Chemistry of Natural and Microbial Products, National Research Centre, Dokki, Cairo 12622, Egypt; ea.mabrouk@nrc.sci.eg; 3 Microbial Chemistry Department, National Research Centre, Dokki, Cairo 12622, Egypt; em.karam@nrc.sci.eg; 4 Department of Pharmaceutical Medicinal Chemistry, Faculty of Pharmacy (Girls), Al-Azhar University, Cairo 11754, Egypt; dremannossier@azhar.edu.eg; 5 Department of Pharmaceutical Chemistry, Faculty of Pharmacy, Taif University, Taif 21974, Saudi Arabia; Marwa_farag@pharm.helwan.edu.eg; 6 Department of Pharmaceutical Chemistry, Faculty of Pharmacy, Helwan University, Cairo 11795, Egypt

**Keywords:** pyridothienopyrimidines, antibacterial activity, enzyme inhibition, DNA gyrase, topoisomerase IV, molecular docking

## Abstract

The growing resistance of bacteria to many antibiotics that have been in use for several decades has generated the need to discover new antibacterial agents with structural features qualifying them to overcome the resistance mechanisms. Thus, novel pyridothienopyrimidine derivatives (**2a**,**b**–**a**,**b**) were synthesized by a series of various reactions, starting with 3-aminothieno[2,3-*b*]pyridine-2-carboxamides (**1a**,**b**). Condensation of compounds **1a**,**b** with cyclohexanone gave 1’*H*-spiro[cyclohexane-1,2’-pyrido[3’,2’:4,5]thieno[3,2-*d*]pyrimidin]-4’(3*’H*)-ones (**2a**,**b**), which in turn were utilized to afford the target 4-substituted derivatives (**3a**,**b**–**8a**,**b**). In vitro antibacterial activity evaluations of all the new compounds (**2a**,**b**–**8a**,**b**) were performed against six strains of Gram-negative and Gram-positive bacteria. The target compounds showed significant antibacterial activity, especially against Gram-negative strains. Moreover, the compounds (**2a**,**b**; **3a**,**b**; **4a**,**b**; and **5a**,**b**) that exhibited potent activity against *Escherichia coli* were selected to screen their inhibitory activity against *Escherichia coli* topoisomerase II (DNA gyrase and topoisomerase IV) enzymes. Compounds **4a** and **4b** showed potent dual inhibition of the two enzymes with IC_50_ values of 3.44 µΜ and 5.77 µΜ against DNA gyrase and 14.46 µΜ and 14.89 µΜ against topoisomerase IV, respectively. In addition, docking studies were carried out to give insight into the binding mode of the tested compounds within the *E. coli* DNA gyrase B active site compared with novobiocin.

## 1. Introduction

Nowadays, the danger of infectious diseases is again on the rise because of the persistent evolution of antibiotic resistance, which will, over time, be a significant threat to health worldwide [1,2].

Antimicrobial-resistant infections are predicted to cause millions of deaths in the coming decades, unless suitable actions are taken to overcome this risk [3]. Thus, there is an urgent need to discover new classes of antimicrobial agents with novel mechanisms of action that can circumvent the resistance problem [4]. Inhibition of bacterial DNA replication enzymes is considered a promising strategy for fighting antimicrobial resistance [5]. Among these enzymes are two bacterial type II topoisomerases, DNA gyrase and topoisomerase IV, which play a significant role in bacterial cell cycle progression [6]. Although the two enzymes have similar structures, each of them has a distinct function during DNA replication: DNA gyrase remains unique in its role to introduce negative supercoiling into DNA, while the critical function of topoisomerase IV is to decatenate daughter chromosomes following DNA replication [6,7].

In clinical use, there are two main classes of antibiotics that target topoisomerase II enzymes, the first is the aminocoumarins class and the second is the quinolones class [8]. Aminocoumarins, such as novobiocin and clorobiocin, inhibit the ATPase domain of the enzymes [9], but their manufacturing and clinical usage have been limited due to their poor pharmacological properties and mammalian cytotoxicity [10]. While, quinolone antibiotics, such as ciprofloxacin and norfloxacin, inhibit topoisomerase II enzymes by binding to a DNA–enzyme complex that leads to stabilizing the DNA double-strand breaks and causes rapid death of the bacterial cell [11,12]. However, after decades of using quinolones in treating a variety of bacterial infections, the number of bacterial strains resistant to this important class of antimicrobial agents has seen an unremitting increase [13]. The most prevalent quinolone-resistance mechanism is associated with specific mutations in the DNA gyrase and/or topoisomerase IV enzymes that reduce the drug’s binding ability to the enzyme–DNA complex, resulting in significant weakness in the quinolone’s therapeutic activity [11,14]. Therefore, some recent efforts to counter microbial resistance mechanisms have included design and synthesis of non-quinolone-based inhibitors able to target varied active sites in bacterial type II topoisomerases and exhibit more potent antimicrobial activity [15,16,17,18].

**Figure 1:**
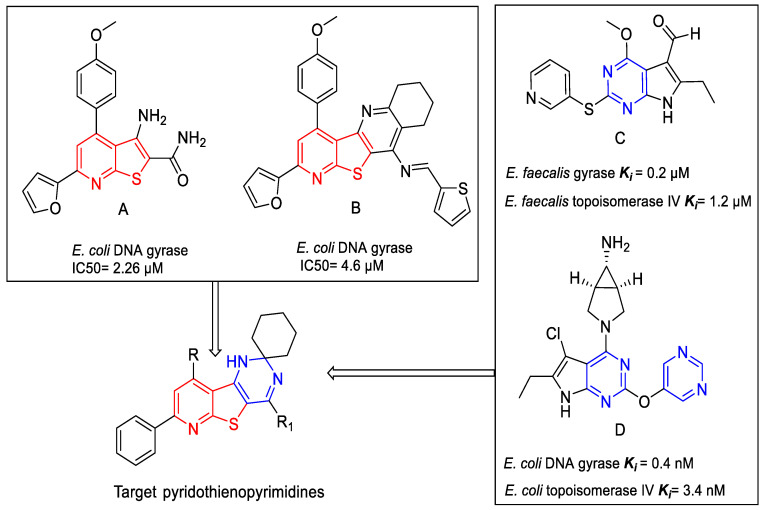
Recently discovered thienopyridines (**A**,**B**) and pyrimidine–based compounds (**C**,**D**) as bacterial type II topoisomerase inhibitors and the design of the target heterocyclic ring system.

On the other hand, many reports have documented the important biological activities of thieno[2,3-*b*]pyridine derivatives in anticancer [19,20,21], antimicrobial [22,23], antiviral [24,25], anti-inflammatory [26] and osteogenic [27] activities. As a result, thieno[2,3-*b*]pyridine compounds and their fused derivatives with the bioactive pyrimidine ring [28,29] have attracted great interest in the last decade; several pyrido[3’,2’:4,5]thieno[3,2-*d*]pyrimidine derivatives were synthesized and gave significant pharmacological properties as antimicrobial [30,31,32], anticancer [33,34] and protein kinase inhibitors [35,36]. Moreover, some thieno[2,3-*b*]pyridine derivatives [37] and pyrimidine-based compounds [38,39] have been discovered in recent years as potent DNA gyrase and/or topoisomerase IV inhibitors, which encourage the design and synthesis of novel thienopyridine-fused pyrimidine compounds to obtain enhanced antibacterial activity (Figure 1).

Based on the above, the current work includes the synthesis of a series of 1’*H*-spiro[cyclohexane-1,2’-pyrido[3’,2’:4,5]thieno[3,2-*d*]pyrimidine] derivatives as new antibacterial agents. The target compounds were linked at position 4 of the pyridothienopyrimidine ring system to different moieties, such as piperazine, morpholine, acetohydrazide and aryl hydrazone, which have valuable pharmacological activities [40,41,42,43]. All the new compounds were screened for their antibacterial activity against different Gram-positive and Gram-negative bacterial strains. Then, the most active compounds against Gram-negative bacteria were tested for their inhibitory activity of *Escherichia coli* DNA gyrase and topoisomerase IV enzymes. Molecular docking studies were also performed for elucidation of the mode of binding of these compounds in the active site of DNA gyrase B kinase.

## 2. Results and Discussion

### 2.1. Chemistry

**Scheme 1:**
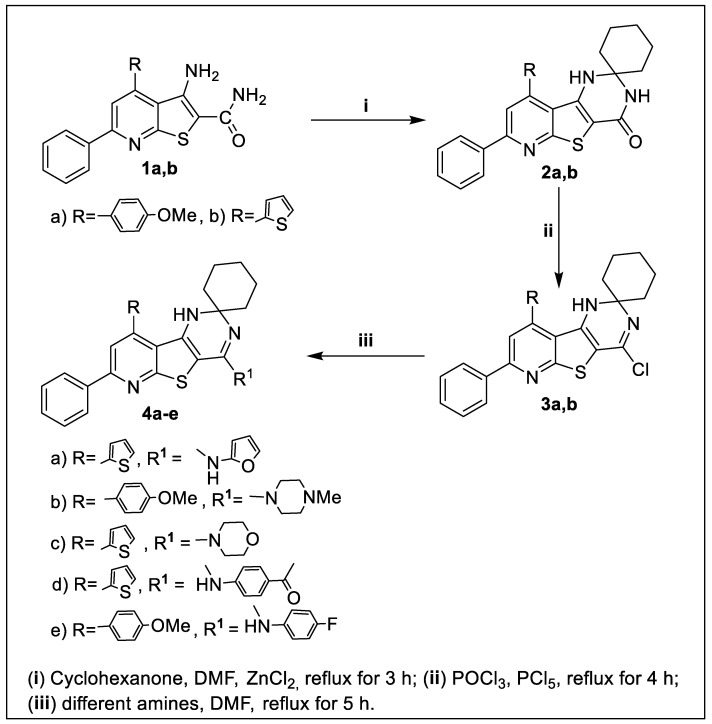
Synthesis of the pyridothienopyrimidine derivatives **2a**,**b**–**4a**–**e**.

**Scheme 2:**
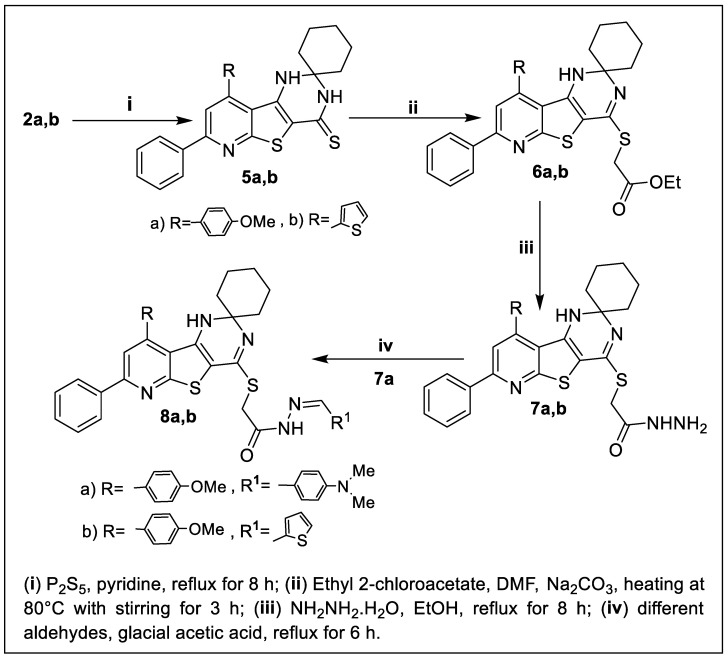
Synthesis of the pyridothienopyrimidine derivatives **5a**,**b**–**8a**,**b**.

The synthesis of the target pyridothienopyrimidines (**2a**,**b**–**8a**,**b**) was achieved via various reactions, as depicted in Scheme 1; Scheme 2. The starting 3-amino-6-phenylthieno[2,3-*b*]pyridine-2-carboxamides **1a**,**b** were prepared [44,45] and underwent a cyclocondensation reaction with cyclohexanone in refluxing *N,N*-dimethyformamide containing anhydrous zinc chloride to produce 1’*H*-spiro[cyclohexane-1,2’-pyrido[3’,2’:4,5]thieno[3,2-*d*]pyrimidin]-4’(3’*H*)-ones **2a**,**b**. The chemical structures of **2a**,**b** were confirmed by their ^1^H NMR spectra, which revealed signals at 1.21–2.02 ppm, corresponding to the 5CH_2_ protons of the spiro cyclohexane ring and two signals corresponding to the 2NH groups of the pyrimidinone ring at 4.53 and 7.90 ppm and 4.90 and 7.88 ppm for **2a** and **2b**, respectively. Furthermore, the ^13^C NMR spectra of **2a**,**b** displayed three signals at 21.6–36.2 ppm of the 5CH_2_ carbons and a signal corresponding to the spiro carbon of **2a** at 69.9 ppm and a signal at 69.8 ppm for that of **2b**. Subsequently, treatment of **2a**,**b** with a refluxing POCl_3_/PCl_5_ mixture gave 4’-chloro derivatives **3a**,**b**. Then the target 1’*H*-spiro[cyclohexane-1,2’-pyrido[3’,2’:4,5]thieno[3,2-*d*]pyrimidin]-4’-amines **4a**–**e** were obtained by carrying out a nucleophilic substitution reaction between 4’-chloro derivatives **3a**,**b** and different amines (furan-2-amine, 1-methylpiperazine, morpholine, 4-acetylaniline and 4-fluoroaniline) in boiling DMF (Scheme 1). In the ^1^H NMR spectrum of **4b**, the protons of the *N*-methylpiperazinyl moiety were verified by three signals at 2.21, 2.59 and 3.75 ppm. The ^13^C NMR spectrum of **4c** also showed the carbons of a morpholine ring as two signals at 45.5 and 66.4 ppm, corresponding to the 2CH_2_N and 2CH_2_O moieties, respectively.

Upon treatment of **2a**,**b** with phosphorus pentasulfide in refluxing pyridine, the pyrido[3’,2’:4,5]thieno[3,2-*d*]pyrimidine]-4’(3’*H*)-thiones **5a**,**b** were formed. IR spectra of **5a**,**b** displayed new bands at 1246.2 cm^−1^ and 1243.2 cm^−1^, corresponding to the C=S groups of **5a** and **5b**, besides the disappearance of the C=O bands of **2a**,**b** at 1658.2 and 1659.4 cm^−1^, respectively. The signal at 181.3 ppm in the ^13^C NMR spectrum of **5a** and at 182.1 ppm in the ^13^C NMR spectrum of **5b** also assisted the presence of the C=S carbon. Subsequent reaction of 4’(3’*H*)-thiones **5a**,**b** with ethyl 2-chloroacete in DMF containing a catalytic amount of anhydrous sodium carbonate afforded the formation of ethyl 2-(pyrido[3’,2’:4,5]thieno[3,2-*d*]pyrimidin-4’-yl)thio)acetates **6a**,**b** via S-alkylation at position 4. The steric hindrance of the bulky spiro cyclohexane moiety was a possible explanation for the forbidding of *N*-alkylation at position 1. ^1^H NMR spectra of **6a** showed a singlet signal at 3.89 ppm of SCH_2_ protons, while the CH_3_ and CH_2_O protons of the ester group were represented by the triplet signal at 1.07 ppm and quartet signal at 4.08 ppm, respectively. The ^13^C NMR spectrum of **6b** also confirmed the occurrence of S-alkylation through the disappearance of the C=S signal at 182.1 ppm, adding to the presence of four new signals at 32.8, 168.7, 61.5 and 14.1 ppm, corresponding to the carbon of SCH_2_ and the three carbons (C=O, OCH_2_ and CH_3_) of the ester group, respectively. Then, the condensation reaction of the esters **6a**,**b** with hydrazine hydrate in refluxing ethanol gave the acetohydrazide derivatives **7a**,**b**. Furthermore, *N’*-(arylidene)-acetohydrazide derivatives **8a**,**b** were obtained by carrying out a nucleophilic addition–elimination reaction between **7a** and an aromatic aldehyde (4-*N*,*N*-dimethylaminobenzaldehyde and/or thiophene-2-carbaldehyde) in refluxing glacial acetic acid (Scheme 2).

In the ^1^H NMR spectrum of **7a**, beside the absence of ester signals, the hydrazide moiety was represented by two signals at 4.62 ppm and 9.18 ppm, corresponding to the NH_2_ and NH groups, respectively. The ^1^H NMR spectrum of *N’*-(4-(dimethylamino)benzylidene derivative **8a** also supported the presence of the arylidine moiety by a signal at 3.18 ppm for the six protons dimethylamino group (N(CH_3_)_2_) and a signal at 8.43 ppm for the proton of the azomethine group (CH=N), as well the signals corresponding to new aromatic protons. Moreover, the ^13^C NMR spectrum of **8a** showed a signal at 44.7 ppm, corresponding to the two carbons of the N(CH_3_)_2_ group. The chemical structures of the new pyridothienopyrimidine derivatives (**2a**,**b**–**8a**,**b**) were confirmed by IR, ^1^H NMR, ^13^C NMR and mass spectra, in addition to the correct results of their elemental microanalyses (Supplementary Materials: NMR spectra of compounds 2a,b–8a,b; Figures S1–S28).

### 2.2. Antibacterial Activity

**Figure 2:**
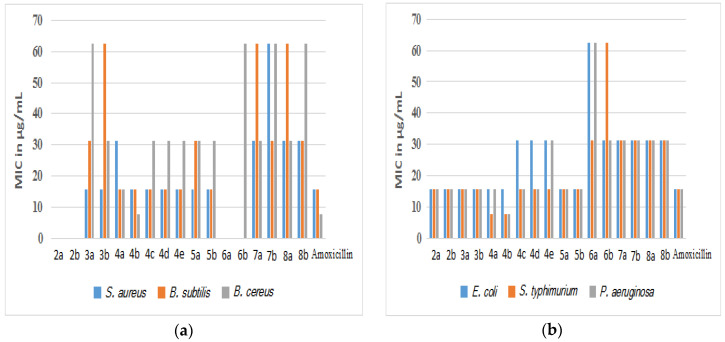
Antibacterial activity (MIC in μg/mL) of the target compounds against (**a**) three strains of Gram-positive bacteria and (**b**) three strains of Gram-negative bacteria.

The results of the in vitro antibacterial activity evaluation (MIC values in μg/mL) of the target pyridothienopyrimidine compounds (**2a**,**b**–**8a**,**b**), listed in Table 1 and Figure 2, revealed the significant activity of the majority of these compounds against the tested Gram-positive bacteria (*Staphylococcus aureus* 25923, *Bacillus cereus* 33018 and *Bacillus subtilis* 6633) and Gram-negative bacteria (*Escherichia coli* 8739*, Salmonella typhimurium* 14028 and *Pseudomonas aeruginosa* 27853) compared with amoxicillin trihydrate as a reference drug. In particular, the activity of the target compounds against the Gram-negative strains were more potent than their activity against the Gram-positive strains. In turn, pyrido[3’,2’:4,5]thieno[3,2-*d*]pyrimidin]-4’(3’*H*)-ones **2a**,**b** showed potent activity against all tested Gram-negative strains (MIC = 15.63 μg/mL), equal to that of the reference drug, but they had no activity against all tested Gram-positive strains at the highest used concentration (125 μg/mL). The 4’-chloro derivatives **3a**,**b** also revealed inhibition activity against Gram-negative strains, the same as amoxicillin, with enhancement in the activity against the Gram-positive strains, especially against *S. aureus* (MIC = 15.63 μg/mL). Further increases in the potency of antibacterial activity against Gram-positive strains were observed in the 4’-amine derivatives **4a**–**e**, especially against *B. subtilis* (MIC = 15.63 μg/mL). The antibacterial activity of **4a**–**e** against the Gram-negative strains varied from potent to moderate, with MIC values ranging from 7.81 to 31.25 μg/mL. Moreover, 4’-(4-methylpiperazin-1-yl) derivative **4b** showed potent activity against all the tested bacterial strains, with MIC values ranging from 7.81 to 15.63 μg/mL, which was equal in potency or more potent than that of amoxicillin. The conversion of 4’(3’*H*)-ones **2a**,**b** to 4’(3’*H*)-thiones **5a**,**b** also enhanced the antimicrobial activity against Gram-positive bacteria and showed inhibition activity ranging from potent to moderate against the three tested microorganisms, with MIC values ranging from 15.63 to 31.25 μg/mL. On the reverse, the esters **6a**,**b** showed dramatic lowering in their activity; they were inactive against the Gram-positive strains and gave moderate or weak activity against the Gram-negative strains (MIC = 31.25 μg/mL and 62.5 μg/mL).

However, the transformation of the esters **6a**,**b** to acetohydrazide derivatives **7a**,**b** and then to their arylidene derivatives **8a**,**b** revealed an improvement in the antibacterial activity against Gram-positive strains, with MIC values ranging from 31.25 to 62.5 μg/mL. Moreover, **7a**,**b** and **8a**,**b** showed moderate activity against all tested Gram-negative strains (MIC = 31.25 μg/mL).

### 2.3. DNA Gyrase and Topoisomerase IV Inhibitory Activity

The target compounds (**2a**,**b**; **3a**,**b**; **4a**,**b**; and **5a**,**b**) that showed the most potent activity against the tested Gram-negative bacteria, especially against *E. coli*, were chosen to evaluate their in vitro inhibitory activity of *E. coli* DNA gyrase and topoisomerase IV. The results of the DNA gyrase supercoiling and topoisomerase IV decatenation assays (IC_50_ values in µΜ) of these compounds are in Table 2, showing the potent dual inhibition of the 4’-amine derivatives **4a**;**b** against both enzymes compared with the two reference inhibitors (ciprofloxacin and novobiocin). The 4’-(4-methyl-piperazin-1-yl)- derivative **4b** was the most potent dual inhibitor with IC_50_ values of 3.44 µΜ against DNA gyrase and 14.46 µΜ against topoisomerase IV, while the IC_50_ values of ciprofloxacin and novobiocin were 3.52 µΜ and 4.19µΜ for DNA gyrase and 17.57 µΜ and 14.59 µΜ for topoisomerase IV, respectively. *N*-(furan-2-yl)- 4’-amine derivative **4a** gave an IC_50_ value of 5.77 µΜ against gyrase and showed a more potent inhibition of topoisomerase IV than that of ciprofloxacin, with an IC_50_ value of 14.89 µΜ. In addition, 4’-chloro derivative **3b** and pyrimidin-4’(3’*H*)-thione derivative **5b** revealed potent inhibitory activity against topoisomerase IV, with IC_50_ values of 17.50 and 17.24µΜ, which were more potent than ciprofloxacin; however, they displayed moderate inhibition to DNA gyrase. The rest of the tested compounds, pyrimidin-4’(3’*H*)-ones **2a**,**b**; 4’-chloro derivative **3a** and pyrimidin-4’(3’*H*)-thione derivative **5a**, showed moderate inhibitory activity against both DNA gyrase and topoisomerase IV, with IC_50_ values ranging from 8.30 to 12.99 µΜ and 21.78 to 23.25 µΜ, respectively.

### 2.4. Molecular Docking Studies

**Figure 3:**
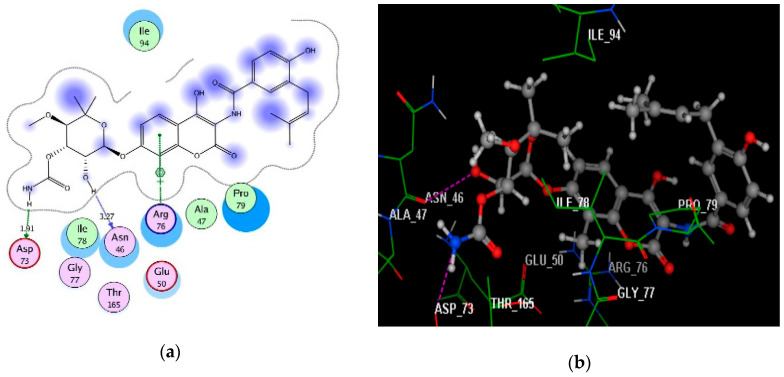
(**a**,**b**) Diagrams illustrating the 2D and 3D binding patterns of novobiocin onto the ATP-active pocket of *E. coli* DNA gyrase B kinase (PDB code: 1AJ6), respectively.

To explore the binding modes of the newly synthesized pyridothienopyrimidines (**2a**,**b**; **3a**,**b**; **4a**,**b**; and **5a**,**b**) with the active site of *E. coli* DNA gyrase B, a molecular docking simulation was accomplished using MOE. Firstly, novobiocin (the original co-crystallized ligand) was re-docked in the active site of *E. coli* DNA gyrase B kinase ((PDB code: 1AJ6) [46,47] (Figure 3) and revealed a score energy of −80 kcal/mol at a root mean square deviation (RMDS) value equal to 0.81 Å. As reported in docking of novobiocin, having a coumarin core linked to oxan-4-yl moiety, the protons of the hydroxyl group of oxan-4-yl and NH_2_ of the carbamate group formed hydrogen bonds within the active site of DNA gyrase B kinase via the backbone of **Asp46** and the side chain of **Asp73**. Furthermore, the coumarin scaffold shared fixation through an arene–cation interaction with the essential amino acid **Arg76** [37,47].

**Figure 4:**
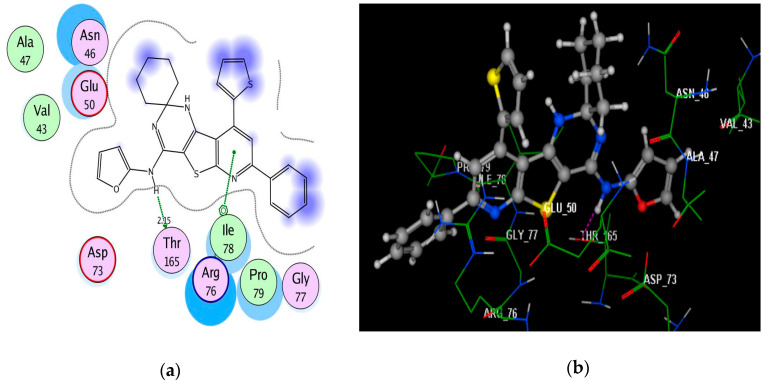
(**a**,**b**) Diagrams illustrating the 2D and 3D binding patterns of compound **4a** onto the ATP-active pocket of *E. coli* DNA gyrase B kinase (PDB code: 1AJ6), respectively.

**Figure 5:**
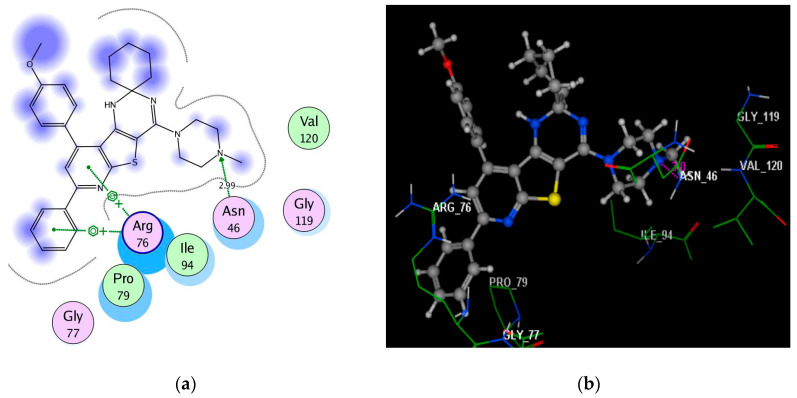
(**a**,**b**) Diagrams illustrating the 2D and 3D binding patterns of compound **4b** onto the ATP-active pocket of *E. coli* DNA gyrase B kinase (PDB code: 1AJ6), respectively.

Then, the target compounds (**2a**,**b**; **3a**,**b**; **4a**,**b**; **5a**,**b**) were docked into the ATP-active sites of *E. coli* DNA gyrase B and the docking results are listed in Table 3. By comparing the energy scores and the binding orientations of the target compounds with that of the original ligand novobiocin, it can be seen that all derivatives displayed promising energy scores ranging, from −5.25 to −6.99 kcal/mol for *E. coli* DNA gyrase B, as well as a noticeable binding affinity between the pyridine scaffold and the essential amino acid **Arg76** via an arene–cation interaction. The 4’-(4-methyl-piperazin-1-yl)- derivative **4b**, which displayed the most potent inhibitory activity, gave the highest binding affinity to DNA gyrase B, with an energy score of −6.99 kcal/mol. Moreover, the *N*-(furan-2-yl)-4’-amine derivative **4a**, which came after **4b** in inhibition potency, displayed the second-best docking score, with a binding energy of −6.64 kcal/mol. Thus, compounds **4a** and **4b** were selected as the ligand examples against the structure of *E. coli* DNA gyrase B (PDB ID code: 1AJ6); the obtained docking models are illustrated in Figure 4; Figure 5.

Regarding to the binding of **4a** with *E. coli* DNA gyrase B, there was an H-bond donor between the NH proton of amino-2-furan scaffold with the side chain of **Thr165** (distance: 2.15 Å), and it exhibited one arene–cation interaction with **Arg76** (Figure 4). However, the pyridine and phenyl moieties of **4b** demonstrated two arene–cation interactions with the key amino acid **Arg76**. Furthermore, the nitrogen of piperazine moiety of **4b** formed with the side chain of **Asn46** a favorable hydrogen bonding (distance: 2.99 Å) (Figure 5).

## 3. Materials and Methods

### 3.1. Chemistry

#### 3.1.1. General Consideration

Melting points were determined by open glass capillary tubes using an Electro thermal IA9100 digital melting point apparatus and were uncorrected. Elemental microanalyses were carried out at the Micro Analytical Unit at Cairo University and were found within ± 0.5%. ^1^H NMR and ^13^C NMR spectra were recorded on a Bruker High Performance Digital FT-NMR Spectrometer Advance III (400/100 MHz) in the presence of TMS as the internal standard. Infrared spectra were recorded by using the KBr disc technique on a Jasco FT/IR-6100, Fourier transform, Infrared spectrometer (Japan) at the cm^−1^ scale. The ESI-mass spectra were measured using an Advion Compact Mass Spectrometer (CMS) NY, USA. Follow-up of the reactions and checking the purity of the compounds were made by TLC on silica gel aluminum sheets (Type 60, F 254, Merck, Darmstadt, Germany) and the spots were illustrated by exposure to UV analysis lamp at λ 254/366 nm or by iodine vapor. The nomenclature of the new synthesized compounds is according to the IUPAC system. The starting 3-amino-6-phenylthieno[2,3-*b*]pyridine-2-carboxamides **1a**,**b** were prepared as per reported methods [44,45].

#### 3.1.2. Synthesis of Pyrido[3’,2’:4,5]Thieno[3,2-*d*]Pyrimidin]-4’(3’*H*)-ones **2a**,**b**

A mixture of compounds **1a**,**b** (0.01 mol) and cyclohexanone (1.47 g, 0.015 mol) in *N,N*-dimethyformamide (20 mL) containing anhydrous ZnCl_2_ (1.36 g, 0.01 mol) was refluxed for 3 h. The reaction mixture was poured onto ice/water and the obtained solid was collected by filtration, washed several times with water and recrystallized from DMF/H_2_O to give compounds **2a**,**b**.

*9’-(4-Methoxyphenyl)-7’-phenyl-1’H-spiro[cyclohexane-1,2’-pyrido[3’,2’:4,5]thieno[3,2-d]pyrimidin]-4’(3’H)-one* (**2a**). It was a yellow powder, yield 81% (3.69 g), m.p. 295−296 °C. Anal. calcd. (%) for C_27_H_25_N_3_O_2_S (455.58): C, 71.18; H, 5.53; N, 9.22; S, 7.04. Found: C, 71.35; H, 5.37; N, 9.43; S, 6.88. ^1^H NMR (DMSO-*d*6, 400 MHz): δ 1.21–1.97 (m, 10H, 5CH_2_), 3.86 (s, 3H, OCH_3_), 4.53 (s, 1H, NH), 7.18 (d, J = 7.2 Hz, 2H, Ar−H), 7.48–7.55 (m, 3H, Ar−H), 7.58 (d, J = 7.2 Hz, 2H, Ar−H), 7.86 (s, 1H, Ar−H), 7.90 (s, 1H, NH), 8.22 (d, J = 6.0 Hz, 2H, Ar−H). ^13^C NMR (DMSO-d_6_, 100 MHz): δ 21.6, 24.5, 36.1 (5CH_2_), 56.0 (OCH_3_), 69.9 (spiro C), 114.5, 119.3, 122.8, 127.6, 129.3, 130.7, 133.6, 135.0, 139.6, 149.8, 154.8, 158.3, 161.5 (Ar−C), 167.9 (C=O). IR (KBr, ν _max_ cm^−1^): 3419.2, 3281.5 (NH), 3047.3, 2924.1, 2856.4 (CH), 1658.2 (C=O). ESI−MS: *m*/*z* = 454.61 [M-H^+^].

*7’-Phenyl-9’-(thiophen-2-yl)-1’H-spiro[cyclohexane-1,2’-pyrido[3’,2’:4,5]thieno[3,2-d]pyrimidin]-4’(3’H)-one* (**2b**). It was a yellow powder, yield 79% (3.40 g), m.p. 289 °C. Anal. calcd. (%) for C_24_H_21_N_3_OS_2_ (431.57): C, 66.79; H, 4.90; N, 9.74; S, 14.86. Found: C, 66.65; H, 5.15; N, 9.49; S, 14.62. ^1^H NMR (DMSO-*d*_6_, 400 MHz): δ 1.21–2.02 (m, 10H, 5CH_2_), 4.90 (s, 1H, NH), 7.34 (dd, *J* = 3.5, 4.7 Hz, 1H, Ar−H), 7.51–7.55 (m, 4H, Ar−H), 7.88 (s, 1H, NH), 7.93–7.98 (m, 2H, Ar−H), 8.22–8.25 (m, 2H, Ar−H). ^13^C NMR (DMSO-*d*_6_, 100 MHz): δ 21.8, 24.6, 36.2 (5CH_2_), 69.8 (spiro C), 119.2, 122.3, 127.7, 128.2, 129.4, 130.1, 133.8, 139.9, 140.4, 145.2, 149.8, 156.0, 158.1 (Ar–C), 167.1 (C=O). IR (KBr, ν _max_ cm^−1^): 3418.6, 3277.2 (NH), 3053.9, 2925.3, 2857.2 (CH), 1659.3 (C=O). ESI−MS: *m*/*z* = 430.48 [M-H^+^].

#### 3.1.3. Synthesis of 4’-Chloropyrido[3’,2’:4,5]Thieno[3,2-*d*]Pyrimidine Derivatives **3a**,**b**

To a solution of compounds **2a**,**b** (5 mmol) in phosphorus oxychloride (15 mL), phosphorus pentachloride (1.04 g, 5 mmol) was added. The reaction mixture was refluxed for 4 h, then left to cool and poured slowly with stirring onto crushed ice. The medium was neutralized with aqueous ammonia (28%) to a pH of 7; the obtained solid was filtered off, washed with water and recrystallized from ethanol to give 4’-chloro derivatives **3a**,**b**.

*4’-Chloro-9’-(4-methoxyphenyl)-7’-phenyl-1’H-spiro[cyclohexane-1,2’-pyrido[3’,2’:4,5]thieno[3,2-d] pyrimidine]* (**3a**). It was a pale-yellow powder, yield 68% (1.61 g), m.p. 201−202 °C. Anal. calcd. (%) for C_27_H_24_ClN_3_OS (474.02): C, 68.41; H, 5.10; N, 8.86; S, 6.76. Found: C, 68.28; H, 5.32; N, 8.59; S, 6.97. ^1^H NMR (DMSO-*d*_6_, 400 MHz): δ 1.28–1.87 (m, 10H, 5CH_2_), 3.85 (s, 3H, OCH_3_), 7.03 (s, 1H, NH), 7.15 (d, *J* = 8.4 Hz, 2H, Ar−H), 7.49–7.54 (m, 3H, Ar−H), 7.74 (d, *J* = 8.4 Hz, 2H, Ar−H), 7.86 (s, 1H, Ar−H), 8.22 (d, *J* = 7.6 Hz, 2H, Ar−H). ^13^C NMR (DMSO-d_6_, 100 MHz): δ 22.1, 24.6, 36.2 (5CH_2_), 56.2 (OCH_3_), 79.7 (spiro C), 114.6, 119.4, 121.2, 123.4, 124.0, 127.6, 129.6, 130.5, 136.7, 139.6, 148.9, 149.7, 155.9, 161.4, 162.4 (Ar−C, C=N). IR (KBr, ν _max_ cm^−1^): 3384.2 (NH), 3058.5, 2934.1, 2848.3 (CH), 1608.6 (C=N), 764.4 (C^_^Cl). ESI−MS: *m*/*z* = 473.0 [M-H^+^].

*4’-Chloro-7’-phenyl-9’-(thiophen-2-yl)-1’H-spiro[cyclohexane-1,2’-pyrido[3’,2’:4,5]thieno[3,2-d]pyrimidine]* (**3b**). It was a buff powder, yield 66% (1.48 g), m.p. 189 °C. Anal. calcd. (%) for C_24_H_20_ClN_3_S_2_ (450.02): C, 64.06; H, 4.48; N, 9.34; S, 14.25. Found: C, 64.25; H, 4.77; N, 9.11; S, 14.53. ^1^H NMR (DMSO-*d*_6_, 400 MHz): δ 1.24–1.87 (m, 10H, 5CH_2_), 6.92 (s, 1H, NH), 7.29 (dd, *J* = 5.3, 8.6 Hz, 1H, Ar−H), 7.50–7.58 (m, 4H, Ar−H), 7.82–7.88 (m, 2H, Ar−H), 8.21-8.27 (m, 2H, Ar−H). ^13^C NMR (DMSO-*d*_6_, 100 MHz): δ 21.5, 24.1, 36.0 (5CH_2_), 79.8 (spiro C), 119.1, 121.7, 123.0, 125.5, 127.5, 129.3, 130.4, 139.7, 141.1, 145.2, 147.5, 156.0, 162.1 (Ar−C, C=N). IR (KBr, ν _max_ cm^−1^): 3367.5 (NH), 3051.2, 2925.1, 2856.3 (CH), 1612.3 (C=N), 762.6 (C^_^Cl). ESI−MS: *m*/*z* = 449.04 [M-H^+^].

#### 3.1.4. Synthesis of Pyrido[3’,2’:4,5]Thieno[3,2-*d*]Pyrimidin]-4’-Amines **4a**–**e**

A mixture of 4’-chloro derivatives **3a**,**b** (1 mmol) and different amines (1 mmol) in *N,N*-dimethy-formamide (20 mL) was heated under reflux for 5 h. After reaction completion, the solvent was evaporated under vacuum and the oily residue was turned to solid by addition of dilute ethanol (50%). The obtained solid was collected by filtration and recrystallized from acetone to give compounds **4a–e**.

*N-(furan-2-yl)-7’-phenyl-9’-(thiophen-2-yl)-1’H-spiro[cyclohexane-1,2’-pyrido[3’,2’:4,5]thieno[3,2-d]pyrimidin]-4’-amine* (**4a**) was synthesized from **3b** (0.45 g, 1 mmol) and furan-2-amine (0.084 g, 1 mmol) according to the general method. It was a brown powder, yield 72% (0.35 g), m.p. 149 °C. Anal. calcd. (%) for C_28_H_24_N_4_OS_2_ (496.65): C, 67.72; H, 4.87; N, 11.28; S, 12.91. Found: C, 67.94; H, 4.61; N, 11.54; S, 12.64. ^1^H NMR (DMSO-*d*_6_, 400 MHz): δ 1.20–2.01 (m, 10H, 5CH_2_), 6.75 (m, 1H, Ar−H), 6.99 (s, 1H, NH), 7.23–7.35 (m, 2H, Ar−H), 7.45–7.58 (m, 4H, Ar−H), 7.81 (s, 1H, Ar−H), 7.91 (d, *J* = 7.2 Hz, 1H, Ar−H), 8.00 (d, *J* =10.4 Hz, 1H, Ar−H), 8.16–8.22 (m, 2H, Ar−H), 8.78 (s, 1H, NH). ^13^C NMR (DMSO-*d*_6_, 100 MHz): δ 21.7, 23.8, 37.3 (5CH_2_), 79.8 (spiro C), 107.8, 109.5, 119.4, 121.7, 123.2, 125.3, 127.4, 128.5, 129.5, 130.2, 139.7, 141.6, 143.8, 144.2, 145.2, 148.1, 155.2, 156.3 (Ar−C, C=N). IR (KBr, ν _max_ cm^−1^): 3432.6, 3366.2 (NH), 3061.1, 2925.2, 2857.4 (CH), 1640.2 (C=N). ESI−MS: *m*/*z* = 495.67 [M-H^+^].

9’-(4-Methoxyphenyl)-4’-(4-methylpiperazin-1-yl)-7’-phenyl-1’H-spiro[cyclohexane-1,2’-pyrido[3’,2’:4,5] thieno[3,2-d]pyrimidine] (**4b**) was synthesized from **3a** (0.47 g, 1 mmol) and 1-methylpiperazine (0.10 g, 1 mmol) according to the general method. It was a beige powder, yield 74% (0.39 g), m.p. 171 °C. Anal. calcd. (%) for C_32_H_35_N_5_OS (537.73): C, 71.48; H, 6.56; N, 13.02; S, 5.96. Found: C, 71.21; H, 6.27; N, 12.74; S, 5.68. ^1^H NMR (DMSO-d_6_, 400 MHz): δ 1.27–1.95 (m, 10H, 5CH_2_), 2.21 (s, 3H, NCH_3_), 2.59 (s, 4H, 2CH_2_N), 3.75 (s, 4H, 2CH_2_N), 3.85 (s, 3H, OCH_3_), 6.99 (s, 1H, NH), 7.13 (d, J = 9.2 Hz, 2H, Ar−H), 7.48–7.53 (m, 3H, Ar−H), 7.73 (d, J = 9.2 Hz, 2H, Ar−H), 7.83 (s, 1H, Ar−H), 8.23 (d, J = 5.2 Hz, 2H, Ar−H). ^13^C NMR (DMSO-d_6_, 100 MHz): δ 21.8, 24.4, 36.4 (5CH_2_), 46.3 (NCH_3_), 48.5 (2CH_2_N), 53.8 (2CH_2_N), 55.7 (OCH_3_), 79.9 (spiro C), 113.2, 119.8, 121.3, 122.9, 124.4, 127.6, 128.9 129.4, 130.1, 136.1, 141.1, 148.9, 149.6, 155.9, 157.2, 161.7 (Ar−C, C=N). IR (KBr, ν _max_ cm^−1^): 3432.5 (NH), 3100.1, 2925.4, 2857.2 (CH), 1640.1 (C=N). ESI−MS: m/z = 536.71 [M-H^+^].

*4-(7’-Phenyl-9’-(thiophen-2-yl)-1’H-spiro[cyclohexane-1,2’-pyrido[3’,2’:4,5]thieno[3,2-d]pyrimidin]-4’-yl)morpholine* (**4c**) was synthesized from **3b** (0.45 g, 1 mmol) and morpholine (0.087 g, 1 mmol) according to the general method. It was a brown powder, yield 76% (0.38 g), m.p. 159–160 °C. Anal. calcd. (%) for C_28_H_28_N_4_OS_2_ (500.68): C, 67.17; H, 5.64; N, 11.19; S, 12.81. Found: C, 66.89; H, 5.31; N, 11.47; S, 12.59. ^1^H NMR (DMSO-*d*_6_, 400 MHz): δ 1.23–2.02 (m, 10H, 5CH_2_), ), 3.06 (s, 4H, 2CH_2_N), 3.79 (s, 4H, 2CH_2_O), 7.04 (s, 1H, NH), 7.33 (s, 1H, Ar−H), 7.51–7.55 (m, 4H, Ar−H), 7.84–7.90 (m, 2H, Ar−H), 8.23 (d, *J* = 10.0 Hz, 2H, Ar−H). ^13^C NMR (DMSO-*d*_6_, 100 MHz): δ 21.1, 24.4, 36.7 (5CH_2_), 45.5 (2CH_2_N), 66.4 (2CH_2_O), 80.1 (spiro C), 119.2, 121.2, 123.4, 125.0, 127.1, 127.5, 128.6, 129.5, 130.2, 139.7, 141.1, 145.7, 149.0, 155.8, 156.8 (Ar−C, C=N). IR (KBr, ν _max_ cm^−1^): 3428.3 (NH), 3107.2, 2922.5, 2852.1 (CH), 1644.6 (C=N). ESI−MS: *m*/*z* = 499.61 [M-H^+^].

1-(4-((7’-phenyl-9’-(thiophen-2-yl)-1’H-spiro[cyclohexane-1,2’-pyrido[3’,2’:4,5]thieno[3,2-d]pyrimidin]-4’-yl)amino)phenyl)ethan-1-one (**4d**) was synthesized from **3b** (0.45 g, 1 mmol) and 4-acetylaniline (0.13 g, 0.001 mol) according to the general method. It was a brown powder, yield 66% (0.36 g), m.p. 141–142 °C. Anal. calcd. (%) for C_32_H_28_N_4_OS_2_ (548.72): C, 70.04; H, 5.14; N, 10.21; S, 11.69. Found: C, 70.33; H, 5.40; N, 10.53; S, 11.43. ^1^H NMR (DMSO-d_6_, 400 MHz): δ 1.21–2.02 (m, 10H, 5CH_2_), 2.66 (s, 3H, CH_3_CO), 6.96 (s, 1H, NH), 7.29–7.35 (m, 1H, Ar−H), 7.43 (d, J = 11.2 Hz, 2H, Ar−H), 7.51–7.55 (m, 4H, Ar−H), 7.77 (d, J = 11.2 Hz, 2H, Ar−H), 7.92–7.96 (m, 2H, Ar−H), 8.21 (d, J = 6.0 Hz, 2H, Ar−H), 10.63 (s, 1H, NH). ^13^C NMR (DMSO-d_6_, 100 MHz): δ 21.8, 24.6, 29.4, 36.2 (5CH_2_, CH_3_), 79.9 (spiro C), 115.8, 119.5, 121.5, 123.1, 125.1, 127.4, 127.7, 128.2, 129.4, 130.2, 132.3, 139.5, 140.9, 142.4, 145.3, 148.9, 150.1, 155.7, 162.8 (Ar−C, C=N), 167.2 (C=O). IR (KBr, ν _max_ cm^−1^): 3416.2, 3280.5 (NH), 3074.3, 2923.2, 2858.1 (CH), 1698.3 (C=O), 1631.4 (C=N). ESI−MS: m/z = 547.69 [M-H^+^].

*N-(4-fluorophenyl)-9’-(4-methoxyphenyl)-7’-phenyl-1’H-spiro[cyclohexane-1,2’-pyrido[3’,2’:4,5]thieno[3,2-d]pyrimidin]-4’-amine* (**4e**) was synthesized from **3a** (0.47 g, 1 mmol) and 4-fluoroaniline (0.11g, 1 mmol) according to the general method. It was a brown powder, yield 67% (0.37 g), m.p. 133–135 °C. Anal. calcd. (%) for C_33_H_29_FN_4_OS (548.68): C, 72.24; H, 5.33; N, 10.21; S, 5.84. Found: C, 72.61; H, 5.64; N, 9.87; S, 5.46. 1H NMR (DMSO-*d*_6_, 400 MHz): δ 1.22–1.84 (m, 10H, 5CH_2_), 3.85 (s, 3H, OCH_3_), 6.78 (d, *J* = 9.6 Hz, 2H, Ar−H), 6.95 (s, 1H, NH), 7.13 (d, *J* = 7.2 Hz, 2H, Ar−H), 7.19 (d, *J* = 9.6 Hz, 2H, Ar−H), 7.48–7.53 (m, 3H, Ar−H), 7.74 (d, *J* = 7.2 Hz, 2H, Ar−H), 7.84 (s, 1H, Ar−H), 8.23 (d, *J* = 8.0 Hz, 2H, Ar−H), 10.26 (s, 1H, NH). IR (KBr, ν _max_ cm^−1^): 3410.2, 3335.4 (NH), 3065.1, 2924.5, 2856.2 (CH), 1628.4 (C=N). ESI−MS: *m*/*z* = 547.64 [M-H^+^].

#### 3.1.5. Synthesis of Pyrido[3’,2’:4,5]Thieno[3,2-*d*]Pyrimidine]-4’(3’*H*)-Thiones **5a**,**b**

A mixture of compounds **2a**,**b** (0.01 mol) and phosphorus pentasulfide (2.22 g, 0.01 mol) in pyridine (30 mL) was refluxed for 8 h. After reaction completion, the reaction solution was poured onto cold water and left in the refrigerator overnight. The formed precipitate was separated by filtration, washed several times with water and recrystallized from CHCl_3_/Pet. ether to give thione derivatives **5a**,**b**.

*9’-(4-Methoxyphenyl)-7’-phenyl-1’H-spiro[cyclohexane-1,2’-pyrido[3’,2’:4,5]thieno[3,2-d]pyrimidine]-4’(3’H)-thione* (**5a**). It was an orange powder, yield 79% (3.72 g), m.p. 254 °C. Anal. calcd. (%) for C_27_H_25_N_3_OS_2_ (471.64): C, 68.76; H, 5.34; N, 8.91; S, 13.60. Found: C, 68.44; H, 5.61; N, 9.19; S, 13.93. ^1^H NMR (DMSO-d6, 400 MHz): δ 1.19–2.03 (m, 10H, 5CH_2_), 3.87 (s, 3H, OCH_3_), 4.80 (s, 1H, NH), 7.17 (d, *J* = 8.5 Hz, 2H, Ar−H), 7.51–7.55 (m, 3H, Ar−H), 7.59 (d, *J* = 8.5 Hz, 2H, Ar−H), 7.84 (s, 1H, Ar−H), 8.22 (d, *J* = 6.5 Hz, 2H, Ar−H), 9.69 (s, 1H, NH). ^13^C NMR (DMSO-d_6_, 100 MHz): δ 21.7, 24.5, 35.7 (5CH_2_), 56.0 (OCH_3_), 70.2 (spiro C), 114.5, 119.4, 121.7, 122.8, 127.6, 128.4, 129.5, 130.2, 134.2, 140.0, 147.8, 149.9, 154.8, 161.5, 163.1 (Ar−C), 181.3 (C=S). IR (KBr, ν _max_ cm^−1^): 3417.5, 3251.4 (NH), 3042.1, 2923.2, 2856.3 (CH), 1246.2 (C=S). ESI−MS: m/z = 470.58 [M - H^+^].

*7’-Phenyl-9’-(thiophen-2-yl)-1’H-spiro[cyclohexane-1,2’-pyrido[3’,2’:4,5]thieno[3,2-d]pyrimidine]-4’(3’H)-thione* (**5b**). It was an orange powder, yield 81% (3.40 g), m.p. 233-234 °C. Anal. calcd. (%) for C_24_H_21_N_3_S_3_ (447.63): C, 64.40; H, 4.73; N, 9.39; S, 21.49. Found: C, 64.78; H, 5.01; N, 9.17; S, 21.71. ^1^H NMR (DMSO-d6, 400 MHz): δ 1.18–2.06 (m, 10H, 5CH_2_), 5.15 (s, 1H, NH), 7.33 (dd, *J* = 3.5, 4.9 Hz, 1H, Ar−H), 7.48–7.57 (m, 4H, Ar−H), 7.92 (s, 1H, Ar−H), 7.93 (s, 1H, Ar−H), 8.21–8.25 (m, 2H, Ar−H), 9.78 (s, 1H, NH). ^13^C NMR (DMSO-d_6_, 100 MHz): δ 21.6, 24.6, 35.3 (5CH_2_), 70.5 (spiro C), 119.4, 121.4, 122.9, 127.7, 128.3, 129.4, 129.7, 130.5, 139.4, 141.3, 145.0, 148.3, 156.0, 162.6 (Ar−C), 182.1 (C=S). IR (KBr, ν _max_ cm^−1^): 3406.3, 3240.4 (NH), 3105.1, 2928.3, 2854.1 (CH), 1243.2 (C=S). ESI−MS: m/z = 446.70 [M - H^+^].

#### 3.1.6. Synthesis of Ethyl 2-(Pyrido[3’,2’:4,5]Thieno[3,2-*d*]Pyrimidin-4-yl-Thio)Acetates **6a**,**b**

A mixture of compounds **5a**,**b** (5 mmol) and ethyl 2-chloroacete (0.60 g, 5 mmol) in *N,N*-dimethylformamide (30 mL) containing anhydrous sodium carbonate (1.0 g) was heated at 80° C with stirring for 3 h. The reaction mixture was poured onto an ice-water mixture and left standing for 1 h. The obtained solid was collected by filtration, washed with water, and recrystallized from ethanol to produce the esters **6a**,**b**.

*Ethyl2-((9’-(4-methoxyphenyl)-7’-phenyl-1’H-spiro[cyclohexane-1,2’-pyrido[3’,2’:4,5]thieno[3,2-d] pyrimidin]-4’-yl)thio)acetate* (**6a**)****. It was an off-white powder, yield 72% (2.0 g), m.p. 275−276 °C. Anal. calcd. (%) for C_31_H_31_N_3_O_3_S_2_ (557.73): C, 66.76; H, 5.60; N, 7.53; S, 11.50. Found: C, 66.43; H, 5.88; N, 7.81; S, 11.17. 1H NMR (DMSO-d6, 400 MHz): δ 1.07 (t, *J* = 10.8 Hz, 3H, C*H_3_*CH_2_), 1.28–1.96 (m, 10H, 5CH_2_), 3.86 (s, 3H, OCH_3_), 3.89 (s, 2H, SCH_2_), 4.08 (q, *J* = 10.8 Hz, 2H, OC*H_2_*CH_3_), 7.05 (s, 1H, NH), 7.14 (d*, J* = 7.6 Hz, 2H, Ar−H), 7.50–7.54 (m, 3H, Ar−H), 7.72 (d, *J* = 7.6 Hz, 2H, Ar−H), 7.89 (s, 1H, Ar−H), 8.23 (d, *J* = 10.0 Hz, 2H, Ar−H). IR (KBr, ν _max_ cm^−1^): 3410.3(NH), 3058.1, 2924.2, 2860.4 (CH), 1735.2 (C=O). ESI−MS: m/z = 556.68 [M - H+].

*Ethyl2-((7’-phenyl-9’-(thiophen-2-yl)-1’H-spiro[cyclohexane-1,2’-pyrido[3’,2’:4,5]thieno[3,2-d] pyrimidin]-4’-yl)thio)acetate* (**6b**). It was a pale-yellow powder, yield 69% (1.84 g), m.p. 261–262 °C. Anal. calcd. (%) for C_28_H_27_N_3_O_2_S_3_ (533.72): C, 63.01; H, 5.10; N, 7.87; S, 18.02. Found: C, 63.29; H, 5.35; N, 7.54; S, 18.33. ^1^H NMR (DMSO-d6, 400 MHz): δ 1.11 (t, *J* = 13.2 Hz, 3H, C*H_3_*CH_2_), 1.28–1.88 (m, 10H, 5CH_2_), 3.90 (s, 2H, SCH_2_), 3.96 (q, *J* = 13.2 Hz, 2H, OC*H_2_*CH_3_), 6.94 (s, 1H, NH), 7.39–7.70 (m, 5H, Ar−H), 7.87–7.95 (m, 2H, Ar−H), 8.23 (m, 2H, Ar−H). ^13^C NMR (DMSO-d_6_, 100 MHz): δ 14.1 (CH_3_), 21.7, 25.5, 32.8, 35.8 (5CH_2_, SCH_2_), 61.5 (CH_2_O), 80.6 (spiro C), 119.2, 122.0, 124.1, 125.6, 127.8, 128.6, 129.5, 130.2, 139.8, 141.1, 144.8, 145.7, 156.4, 163.1 (Ar−C, C=N), 168.7 (C=O). IR (KBr, ν _max_ cm^−1^): 3417.2 (NH), 3068.0, 2926.4, 2863.2 (CH), 1736.5 (C=O). ESI−MS: m/z = 532.76 [M - H^+^].

#### 3.1.7. Synthesis of Pyrido[3’,2’:4,5] Thieno[3,2-*d*]Pyrimidin]-4’-yl)thio)Acetohydrazides **7a**,**b**

A mixture of **6a**,**b** (0.004 mol) and hydrazine hydrate 98% (1mL, excess) in absolute ethanol (50 mL) was refluxed for 8 h. After reaction completion, the obtained solid was collected by filtration and recrystallized from DMF/H_2_O to give acetohydrazide derivatives **7a**,**b**.

2-((9’-(4-methoxyphenyl)-7’-phenyl-1’H-spiro[cyclohexane-1,2’-pyrido[3’,2’:4,5]thieno[3,2-d]pyrimidin]-4’-yl)thio)acetohydrazide (**7a**). It was a red powder, yield 66% (1.43 g), m.p. 234 °C. Anal. calcd. (%) for C_29_H_29_N_5_O_2_S_2_ (543.70): C, 64.06; H, 5.38; N, 12.88; S, 11.79. Found: C, 64.33; H, 5.65; N, 13.16; S, 11.47. ^1^H NMR (DMSO-d6, 400 MHz): δ 1.26–1.99 (m, 10H, 5CH_2_), 3.86 (s, 3H, OCH_3_), 3.89 (s, 2H, SCH_2_), 4.62 (s, 2H, NH_2_), 7.05 (s, 1H, NH), 7.17 (d, J = 8.6 Hz, 2H, Ar−H), 7.49–7.59 (m, 3H, Ar−H), 7.74 (d, J = 8.6 Hz, 2H, Ar−H), 7.84 (s, 1H, Ar−H), 8.27 (d, J = 7.0 Hz, 2H, Ar−H), 9.18 (s, 1H, NH). IR (KBr, ν _max_ cm^−1^): 3410.3, 3320.3, 3240.1(NH), 3057.5, 2927.2, 2858.4 (CH), 1685.3 (C=O). ESI−MS: m/z = 542.73 [M - H^+^].

*2-((7’-phenyl-9’-(thiophen-2-yl)-1’H-spiro[cyclohexane-1,2’-pyrido[3’,2’:4,5]thieno[3,2-d]pyrimidin]-4’-yl)thio)acetohydrazide* (**7b**). It was a red powder, yield 62% (1.29 g), m.p. 222 °C. Anal. calcd. (%) for C_26_H_25_N_5_OS_3_ (519.70): C, 60.09; H, 4.85; N, 13.48; S, 18.51. Found: C, 60.48; H, 5.19; N, 13.11; S, 18.19. ^1^H NMR (DMSO-d6, 400 MHz): δ 1.27–1.96 (m, 10H, 5CH_2_), 3.87 (s, 2H, SCH_2_), 4.59 (s, 2H, NH_2_), 6.99 (s, 1H, NH), 7.32–7.74 (m, 5H, Ar−H), 7.86–7.91 (m, 2H, Ar−H), 8.26 (m, 2H, Ar−H), 9.22 (s, 1H, NH). IR (KBr, ν _max_ cm^−1^): 3414.3, 3326.1, 3251.4 (NH), 3061.1, 2924.3, 2857.2 (CH), 1680.1 (C=O). ESI−MS: m/z = 518.66 [M - H^+^].

#### 3.1.8. Synthesis of *N*’-(Arylidene)-2-(Pyrido[3’,2’:4,5]Thieno[3,2-*d*] Pyrimidin]-4’-yl)Thio) Acetohydrazides **8a**,**b**

A mixture of **7a** (0.54 g, 1 mmol) and different aldehydes, namely, 4-(dimethylamino)benzaldehyde and thiophene-2-carbaldehyde (1 mmol) in glacial acetic acid (20 mL), was refluxed for 6 h. After reaction completion, the reaction mixture was concentrated and poured onto cold water. The formed solid was collected by filtration, washed with water and recrystallized from ethanol to give compounds **8a**,**b**.

N’-(4-(dimethylamino)benzylidene)-2-((9’-(4-methoxyphenyl)-7’-phenyl-1’H-spiro[cyclohexane-1,2’-pyrido[3’,2’:4,5]thieno[3,2-d]pyrimidin]-4’-yl)thio)acetohydrazide (**8a**)****. It was a brown powder, yield 61% (0.41 g), m.p. 199–200 °C. Anal. calcd. (%) for C_38_H_38_N_6_O_2_S_2_ (674.88): C, 67.63; H, 5.68; N, 12.45; S, 9.50. Found: C, 67.31; H, 5.93; N, 12.69; S, 9.22. ^1^H NMR (DMSO-d6, 400 MHz): δ 1.23–1.92 (m, 10H, 5CH_2_), 3.01 (s, 6H, N(CH_3_)_2_), 3.85 (s, 3H, OCH_3_), 3.98 (s, 2H, SCH_2_), 6.97 (s, 1H, NH), 7.16 (d, J = 8.4 Hz, 2H, Ar−H), 7.22 (d, J = 9.6 Hz, 2H, Ar−H), 7.54–7.55 (m, 3H, Ar−H), 7.74 (d, J = 8.4 Hz, 2H, Ar−H), 7.87 (s, 1H, Ar−H), 7.94 (d, J = 9.6 Hz, 2H, Ar−H), 8.26 (d, J = 7.2 Hz, 2H, Ar−H), 8.43 (s, 1H, CH=N), 11.14 (s, 1H, NH). ^13^C NMR (DMSO-d_6_, 100 MHz): δ 21.6, 24.4, 33.6, 36.5 (5CH_2_, SCH_2_), 44.7 (N(CH_3_)_2_), 56.0 (OCH_3_), 80.3 (spiro C), 112.1, 114.6, 119.5, 121.6, 122.9, 124.5, 127.8, 128.2, 129.1, 129.9, 130.8, 134.7, 139.9, 145.1, 148.0, 150.1, 154.0, 155.2, 161.5, 163.6 (Ar−C, CH=N), 169.9 (C=O). IR (KBr, ν _max_ cm^−1^): 3417.1, 3325.5 (NH), 3066.2, 2924.2, 2853.3 (CH), 1678.1 (C=O). ESI−MS: m/z = 673.91 [M - H^+^].

2-((9’-(4-methoxyphenyl)-7’-phenyl-1’H-spiro[cyclohexane-1,2’-pyrido[3’,2’:4,5]thieno[3,2-d]pyrimidin]-4’-yl)thio)-N’-(thiophen-2-ylmethylene)acetohydrazide (**8b**)****. It was a buff powder, yield 63% (0.40 g), m.p. 178–179 °C. Anal. calcd. (%) for C_34_H_31_N_5_O_2_S_3_ (637.84): C, 64.03; H, 4.90; N, 10.98; S, 15.08. Found: C, 64.35; H, 5.17; N, 10.66; S, 15.36. ^1^H NMR (DMSO-d6, 400 MHz): δ 1.28–1.96 (m, 10H, 5CH_2_), 3.87 (s, 3H, OCH_3_), 3.98 (s, 2H, SCH_2_), 6.98 (s, H, NH), 7.15 (d, J = 10.8 Hz, 2H, Ar−H), 7.39–7.98 (m, 9H, Ar−H), 8.26 (d, J = 9.6 Hz, 2H, Ar−H), 8.53 (s, 1H, CH=N), 10.96 (s, 1H, NH). IR (KBr, ν _max_ cm^−1^): 3412.3, 3310.2 (NH), 3069.1, 2925.2, 2857.4 (CH), 1680.5 (C=O). ESI−MS: m/z = 636.78 [M - H^+^].

### 3.2. In Vitro Antibacterial Screening

All the newly synthesized compounds (**2a**,**b**–**8a**,**b**) and the standard drug (amoxicillin trihydrate) were evaluated for their antibacterial activity against three strains of Gram-positive bacteria (*S. aureus* 25923, *B. cereus* 33018 and *B. subtilis* 6633) and three strains of Gram-negative bacteria (*E. coli* 8739*, S. typhimurium* 14028 and *P. aeruginosa* 27853) by using the broth dilution method [48]. A twofold serial dilution was performed to obtain the required concentrations (125, 62.5, 31.25, 15.63, 7.81, 3.91, 1.95 and 0.97, μg/mL) for the target compounds and the reference drug. The minimum concentration of the sample that showed no growth of the tested microorganism (MIC) were specified; then the MIC values for the compounds and standard drug were listed (see Table 1).

### 3.3. DNA Gyrase Supercoiling and Topoisomerase IV Decatenation Inhibition Assays

Eight of the target compounds (**2a**, **2b**, **3a**, **3b**, **4a**, **4b**, **5a**, and **5b**) were chosen to evaluate their inhibitory activity against bacterial type II topoisomerase enzymes. The assay kits of *E. coli* DNA gyrase supercoiling and *E. coli* topoisomerase IV decatenation were provided by TopoGEN, Inc. (Port Orange, FL) and the assays were performed according to established protocols obtained from the supplier [49]. The new compounds and two standard inhibitors (ciprofloxacin and novobiocin) were dissolved in DMSO and serially diluted at concentrations of 100, 10, 1 and 0.1 μM, and then assayed in reaction mixtures in three different replicate runs. For DNA gyrase supercoiling: the final reaction volume was 20 µL, which included 35 mM Tris pH 7, 2 mM DTT, 24 mM KCl, 4 mM MgCl_2_, 1.8 mM spermidine, 0.1 mg/mL acetylated BSA, 6.5% (*w*/*v*) glycerol, 1 mM ATP, 0.1 mg/mL album and 0.2 mg pBR322 substrate. While, for topoisomerase IV decatenation: the final reaction volume was 20 µL, containing 40 mM Tris pH 7.5, 10 mM DTT, 6 mM MgCl_2_, 100 mM potassium glutamate, 1 mM ATP, 50 mg/mL acetylated BSA and 0.2 mg kDNA substrate. The reactions were initiated by addition of 2 U of *E.coli* DNA gyrase or *E.coli* topoisomerase IV (TopoGen), and 3 µL of inhibitor solution in 10% DMSO, and then were incubated with shaking for 30 min at 37 °C. All of the reactions were terminated by the addition of 10 mL of a 3X gel-loading buffer (final concentration: 6 mM EDTA, 1.2% SDS, 0.02% bromophenol blue, and 10% glycerol blue), after which 20 mL of this was loaded on a 1% agarose, TAE (0.01 M EDTA pH 8.3, 40 mM Tris-acetate) gel that was then run at 60 V for 3 h. The gel was stained by (0.5 mg/L) ethidium bromide in TAE for 30 min and then de-stained in water for 20 min. Fluorescent images were taken at a wavelength of 300 nm on a UV transilluminator imaging system. The fluorescence intensity of the supercoiled plasmid reaction product and the decatenation product, in the case of gyrase and topo IV, were quantitated using ImagQuant software (Molecular Dynamics, Sunnyvale, CA, USA). The results as IC50 values (concentration of the tested compound that leads to 50% inhibition of enzyme activity) for all samples were determined by nonlinear regression analysis in GraphPad Prism [49,50]. The average IC_50_ values (μM) of the triplicate experiments were calculated for the target compounds and the two reference antibiotics and then listed in Table 2.

### 3.4. Molecular Docking Study

The molecular docking simulation study was done using Molecular Operating Environment (MOE^®^) 2008.10 software [51]. The crystal structures of *E. coli* DNA gyrase B complexed with their ligand novobiocin (PDB codes: 1AJ6 and 1S14) [46,47] were retrieved from the Protein Data Bank. At the beginning, the co-crystallized ligand was re-docked into the assigned active *E. coli* DNA gyrase B enzyme to evaluate the root mean square deviation value. Then, the molecular docking procedure was performed for the newly synthesized compounds (**2a**,**b**; **3a**,**b**; **4a**,**b**; **5a**,**b**) into the ATP-binding site of *E. coli* DNA gyrase B (PDB code: 1AJ6 and 1S14), following the reported method [37].

## 4. Conclusions

This work included synthesis of novel 4-substituted-1*’H*-spiro[cyclohexane-1,2’-pyrido[3’,2’:4,5] thieno[3,2-*d*]pyrimidin] compounds (**2a**,**b**–**8a**,**b**) and the in vitro evaluation of these compounds against six strains of both Gram-positive and Gram-negative bacteria compared with amoxicillin trihydrate as a reference drug. The tested pyridothienopyrimidine compounds (**2a**,**b**–**8a**,**b**) showed significant antibacterial activity, especially against Gram-negative strains, with MIC values ranging from 7.81 to 62.5 μg/mL. Compounds **2a**,**b**; **3a**,**b**; and **5a**,**b** gave potent activity against all tested Gram-negative bacteria, equal to that of the reference drug, with an MIC value of 15.63 μg/mL. The *N*-(furan-2-yl)-4’-amine derivative **4a** also displayed potent activity against all Gram-negative strains, besides its potent to moderate activity against Gram-positive strains. Moreover, 4’-(4-methyl-piperazin-1-yl)- derivative **4b** was the most active compound compared with the reference drug; it gave potent activity against all the tested bacterial strains, with MIC values ranging from 7.81 to 15.63 μg/mL. In turn, the antibacterial activity of compounds **6a**,**b**; **7a**,**b** and **8a**,**b** varied from weak, with an MIC value of 62.5 μg/mL, to moderate, with an MIC value of 31.25μg/mL, against the tested microorganisms. Thus, compounds **2a**,**b**; **3a**,**b**; **4a**,**b** and **5a**,**b**, because they were the most potent compounds against *E. coli,* were selected to evaluate their in vitro inhibitory activity of *E. coli* topoisomerase II enzymes (DNA gyrase and topoisomerase IV). The tested compounds showed dual inhibition of the two enzymes and their inhibitory activity varied from potent to moderate compared with the two reference antibiotics (ciprofloxacin and novobiocin). Furthermore, compound **4b** displayed dual inhibition, and was more potent than the two references, with IC_50_ values of 3.44 µΜ and 14.46 µΜ against DNA gyrase and topoisomerase IV, respectively. Furthermore, **4a** came after **4b** in inhibition potency with IC_50_ values of 5.77 µΜ for DNA gyrase and 14.89 µΜ for topoisomerase IV. In addition, docking studies were performed with compounds **2a**,**b**; **3a**,**b**; **4a**,**b** and **5a**,**b**, to illustrate their binding mode in the active site of DNA gyrase B compared with that of novobiocin. The docking results of the tested compounds were compatible with their inhibitory potency and gave binding scores ranging from −5.25 to −6.99 kcal/mol.

The results of this study pointed to the importance of pyridothienopyrimidine compounds as a promising heterocyclic sector that, with further study and development, can provide new antimicrobial agents competent of facing the increasing antimicrobial resistance.

## Figures and Tables

**Table 1:** Minimum Inhibitory Concentration (MIC) values in μg/mL of all the target compounds against different bacterial strains.

Compound	Gram-Positive Bacteria			Gram-Negative Bacteria		
	*S. aureus*	*B. subtilis*	*B. cereus*	*E. coli*	*S. typhimurium*	*P. aeruginosa*
**2a**	>125	>125	>125	15.63	15.63	15.63
**2b**	>125	>125	>125	15.63	15.63	15.63
**3a**	15.63	31.25	62.5	15.63	15.63	15.63
**3b**	15.63	62.5	31.25	15.63	15.63	15.63
**4a**	31.25	15.63	15.63	15.63	7.81	15.63
**4b**	15.63	15.63	7.81	15.63	7.81	7.81
**4c**	15.63	15.63	31.25	31.25	15.63	15.63
**4d**	15.63	15.63	31.25	31.25	15.63	15.63
**4e**	15.63	15.63	31.25	31.25	15.63	31.25
**5a**	15.63	31.25	31.25	15.63	15.63	15.63
**5b**	15.63	15.63	31.25	15.63	15.63	15.63
**6a**	>125	>125	>125	62.5	31.25	62.5
**6b**	>125	>125	62.5	31.25	62.5	31.25
**7a**	31.25	62.5	31.25	31.25	31.25	31.25
**7b**	62.5	31.25	62.5	31.25	31.25	31.25
**8a**	31.25	62.5	31.25	31.25	31.25	31.25
**8b**	31.25	31.25	62.5	31.25	31.25	31.25
**Amoxicillin**	15.63	15.63	7.81	15.63	15.63	15.63

**Table 2:**
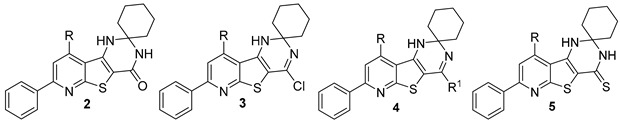
Inhibitory activity of some selected compounds against *E. coli* DNA gyrase and topoisomerase IV enzymes.

			IC_50_(μM)	
Compound	R	R^1^	DNA Gyrase Supercoiling	Topoisomerase IV Decatenation
**2a**	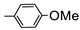		8.30	21.99
**2b**	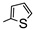		10.42	22.03
**3a**	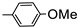		8.99	21.78
**3b**	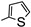		6.96	17.50
**4a**	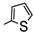	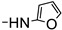	5.77	14.89
**4b**	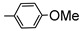	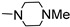	3.44	14.46
**5a**	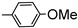		12.99	23.25
**5b-**	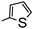		14.23	17.24
**Ciprofloxacin**			3.52	17.57
**Novobiocin**			4.19	14.59

**Table 3:** Docking results of compounds **2a**,**b**; **3a**,**b**; **4a**,**b**; and **5a**,**b** with *E. coli* DNA gyrase B kinase using MOE software version 2008.10.

Compound No.	Docking Score (Kcal/mol)	Amino Acid Residues (Bond Length A^o^)	Atoms of Compound	Type of Bond
**Novobiocin**	−6.80	Asn46(3.27); Asp73(1.91); Arg76	H(OH)(oxan-4-yl); H(OCONH_2_); C_6_H_2_(coumarin)	H-don H-don Arene–cation
**2a**	−5.25	Arg76; Gly77(2.79)	pyridine; O(pyrimidone)	Arene–cation H-acc
**2b**	−5.74	Arg76; Gly77(2.25)	pyridine; O(pyrimidone)	Arene–cation H-acc
**3a**	−5.65	Arg76; Thr165(2.50)	pyridine; N-3(pyrimidine)	Arene–cation H-acc
**3b**	−5.90	Arg76; Thr165(2.27)	pyridine; N-3(pyrimidine)	Arene–cation H-acc
**4a**	−6.64	Arg76; Thr165(2.15)	pyridine; H(NH-furan-2-yl)	Arene–cation H-don
**4b**	−6.99	Asn46(2.99); Arg76; Arg76	N(piperazine); pyridine; phenyl	H-acc Arene–cation Arene–cation
**5a**	−5.30	Arg76	pyridine	Arene–cation
**5b**	−5.55	Arg76	pyridine	Arene–cation

## Supplementary Materials

The following are available online at https://www.mdpi.com/2079-6382/9/10/695/s1, Figures S1–S28: NMR spectra of compounds **2a**,**b**–**8a**,**b**.
