# Supplementary material for: Synthesis and Biological Evaluation of New Pyridothienopyrimidine Derivatives as Antibacterial Agents and Escherichia coli Topoisomerase II Inhibitors
Source: Antibiotics (Basel). 2020 Oct 14;9(10):695. doi: 10.3390/antibiotics9100695 (PMC7602199; doi:10.3390/antibiotics9100695)
Supplement: Supplementary file 1 [file antibiotics-09-00695-s001.pdf]

Supplementary Materials

Synthesis and Biological Evaluation of New Pyridothienopyrimidine Derivatives as Antibacterial Agents and *Escherichia coli* Topoisomerase II Inhibitors

Eman M. Mohi El-Deen [1,*], Eman A. Abd El-Meguid [2], Eman A. Karam [3], Eman S. Nossier [4] and Marwa F. Ahmed [5,6]

[1] Department of Therapeutic Chemistry, National Research Centre, Dokki, Cairo, 12622 Egypt.
[2] Department of Chemistry of Natural and Microbial Products, National Research Centre, Dokki, Cairo, 12622 Egypt.
[3] Microbial Chemistry Department, National Research Centre, Dokki, Cairo, 12622 Egypt.
[4] Department of Pharmaceutical Medicinal Chemistry, Faculty of Pharmacy (Girls), Al-Azhar University, Cairo 11754, Egypt.
[5] Department of Pharmaceutical Chemistry, Faculty of Pharmacy, Taif University, Taif 21974, Kingdom of Saudi Arabia.
[6] Department of Pharmaceutical Chemistry, Faculty of Pharmacy, Helwan University, Cairo, 11795, Egypt.
* Correspondence: e.mohi.2010@live.com; Tel.: +20-0106-385-3338.

Table of contents

	Page
Figure S1. ^{1}H-NMR (400 MHz, DMSO-d_6) spectrum of **2a**.	S3
Figure S2. ^{13}C-NMR (100 MHz, DMSO- d_6) spectrum of **2a**.	S3
Figure S3. ^{1}H-NMR (400 MHz, DMSO-d_6) spectrum of **2b**.	S4
Figure S4. ^{13}C-NMR (100 MHz, DMSO- d_6) spectrum of **2b**.	S4
Figure S5. ^{1}H-NMR (400 MHz, DMSO- d_6) spectrum of **3a**.	S5
Figure S6. ^{13}C-NMR (100 MHz, DMSO- d_6) spectrum of **3a**.	S5
Figure S7. ^{1}H-NMR (400 MHz, DMSO- d_6) spectrum of **3b**.	S6
Figure S8. ^{13}C-NMR (100 MHz, DMSO- d_6) spectrum of **3b**.	S6
Figure S9. ^{1}H-NMR (400 MHz, DMSO- d_6) spectrum of **4a**.	S7
Figure S10. ^{13}C-NMR (100 MHz, DMSO- d_6) spectrum of **4a**.	S7
Figure S11. ^{1}H-NMR (400 MHz, DMSO- d_6) spectrum of **4b**.	S8

Figure S12. ^{13}C-NMR (100 MHz, DMSO- d_6) spectrum of **4b**. S8

Figure S13. ^{1}H-NMR (400 MHz, DMSO- d_6) spectrum of **4c**. S9

Figure S14. ^{13}C-NMR (100 MHz, DMSO- d_6) spectrum of **4c**. S9

Figure S15. ^{1}H-NMR (400 MHz, DMSO- d_6) spectrum of **4d**. S10

Figure S16. ^{13}C-NMR (100 MHz, DMSO- d_6) spectrum of **4d**. S10

Figure S17. ^{1}H-NMR (400 MHz, DMSO- d_6) spectrum of **4e**. S11

Figure S18. ^{1}H-NMR (400 MHz, DMSO- d_6) spectrum of **5a**. S11

Figure S19. ^{13}C-NMR (100 MHz, DMSO- d_6) spectrum of **5a**. S12

Figure S20. ^{1}H-NMR (400 MHz, DMSO- d_6) spectrum of **5b**. S12

Figure S21.^{13}C-NMR (100 MHz, DMSO- d_6) spectrum of **5b**. S13

Figure S22. ^{1}H-NMR (400 MHz, DMSO- d_6) spectrum of **6a**. S13

Figure S23. ^{1}H-NMR (400 MHz, DMSO- d_6) spectrum of **6b**. S14

Figure S24. ^{13}C-NMR (100 MHz, DMSO- d_6) spectrum of **6b**. S14

Figure S25. ^{1}H-NMR (400 MHz, DMSO- d_6) spectrum of **7a**. S15

Figure S26. ^{1}H-NMR (400 MHz, DMSO- d_6) spectrum of **8a**. S15

Figure S27. ^{13}C-NMR (100 MHz, DMSO- d_6) spectrum of **8a**. S16

Figure S28. ^{1}H-NMR (400 MHz, DMSO- d_6) spectrum of **8b**. S16

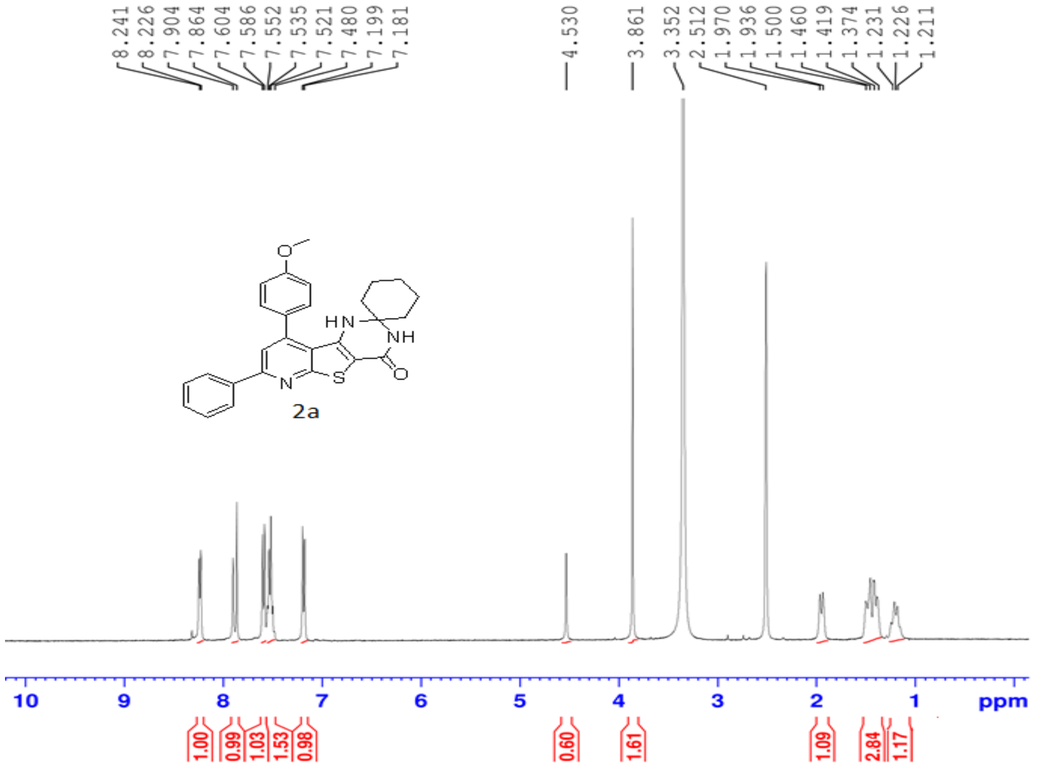

Fig. S1 ^{1}H NMR (400 MHz) in DMSO-d_6 of compound **2a**

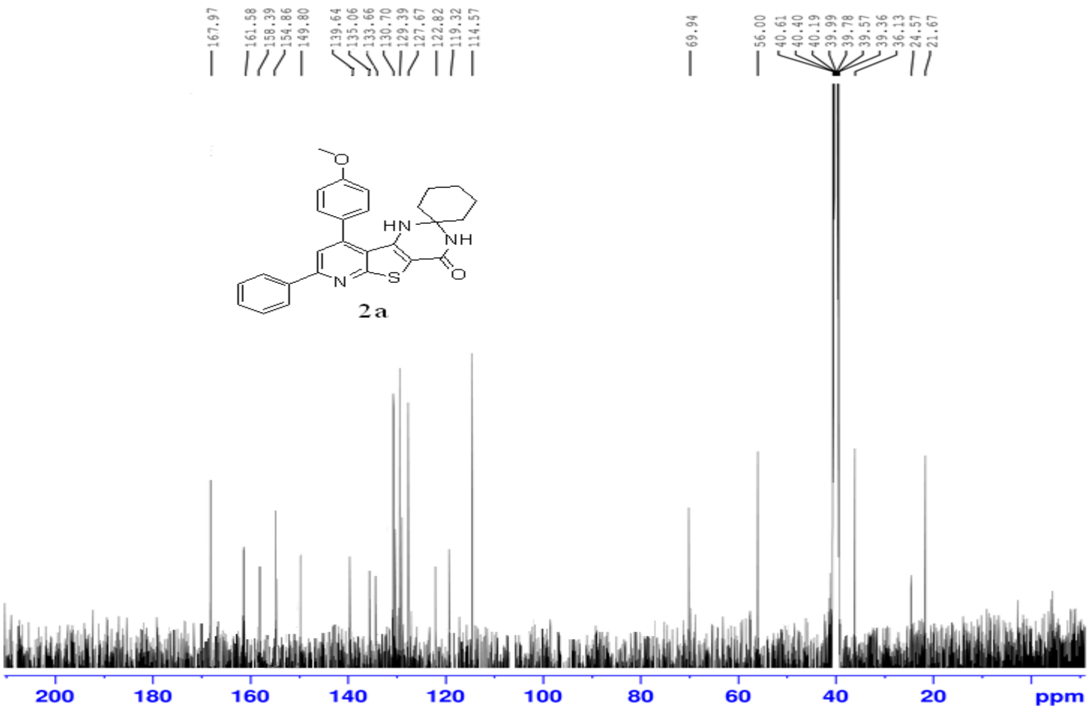

Fig. S2 ^{13}C NMR (100 MHz) in DMSO-d_6 of compound **2a**

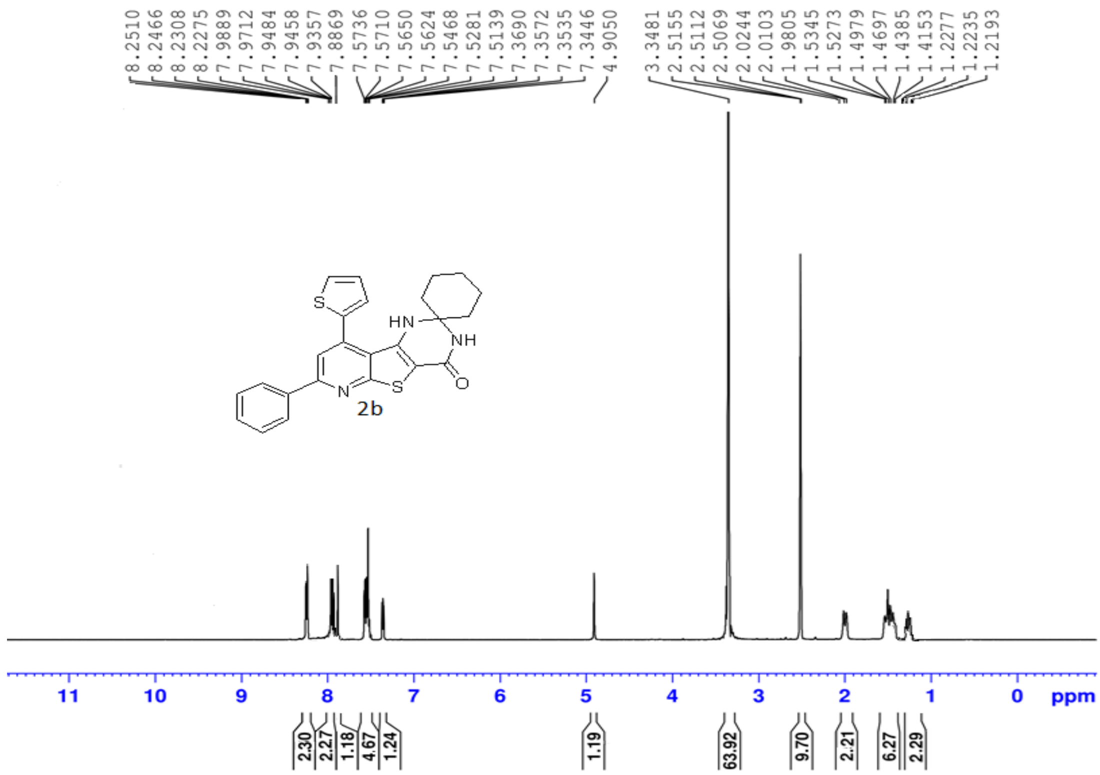

Fig. S3 ^{1}H NMR (400 MHz) in DMSO-d_6 of compound **2b**

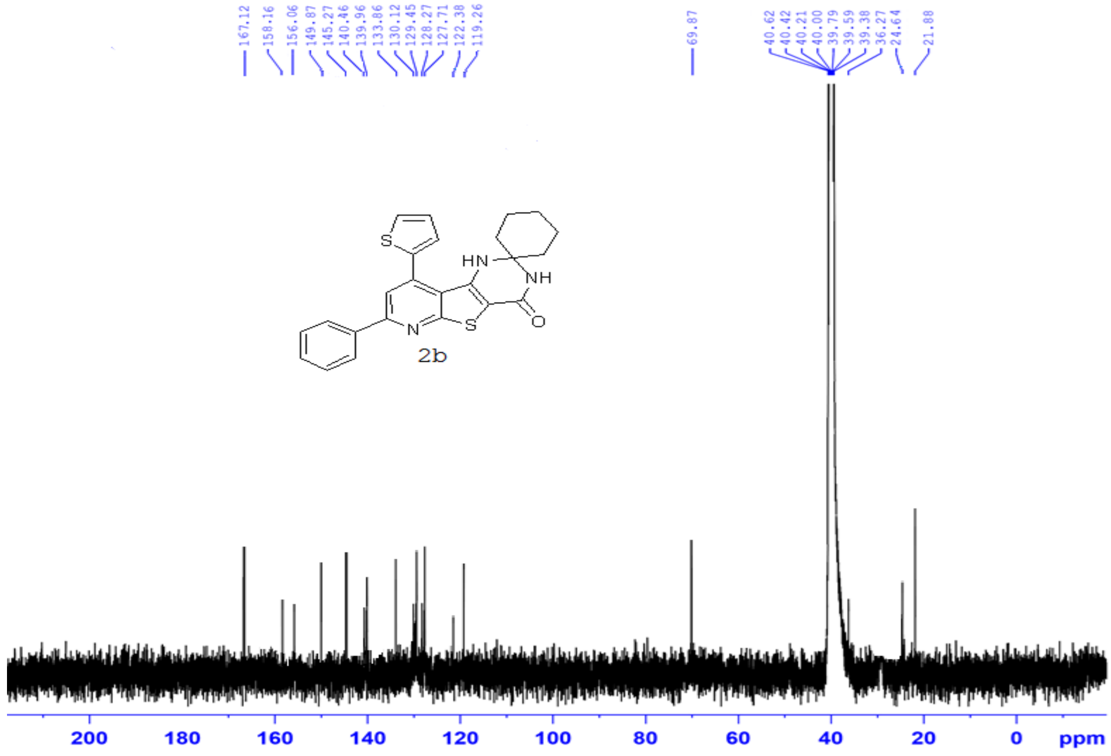

Fig. S4 ^{13}C NMR (100 MHz) in DMSO-d_6 of compound **2b**

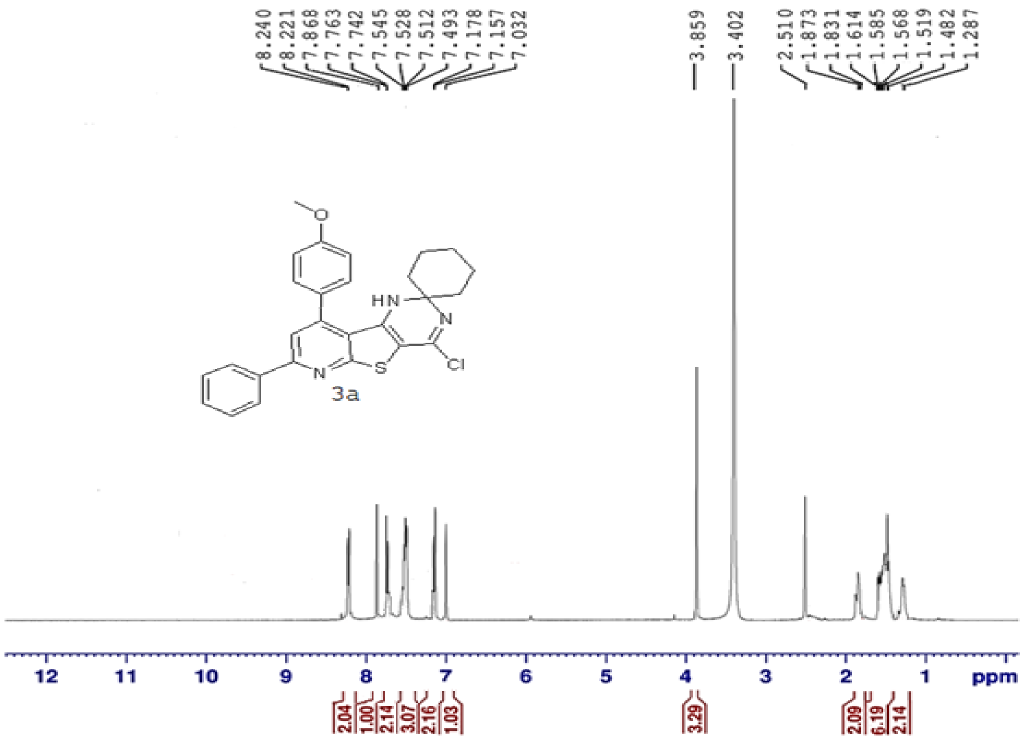

Fig. S5 ^{1}H NMR (400 MHz) in DMSO-d_6 of compound **3a**

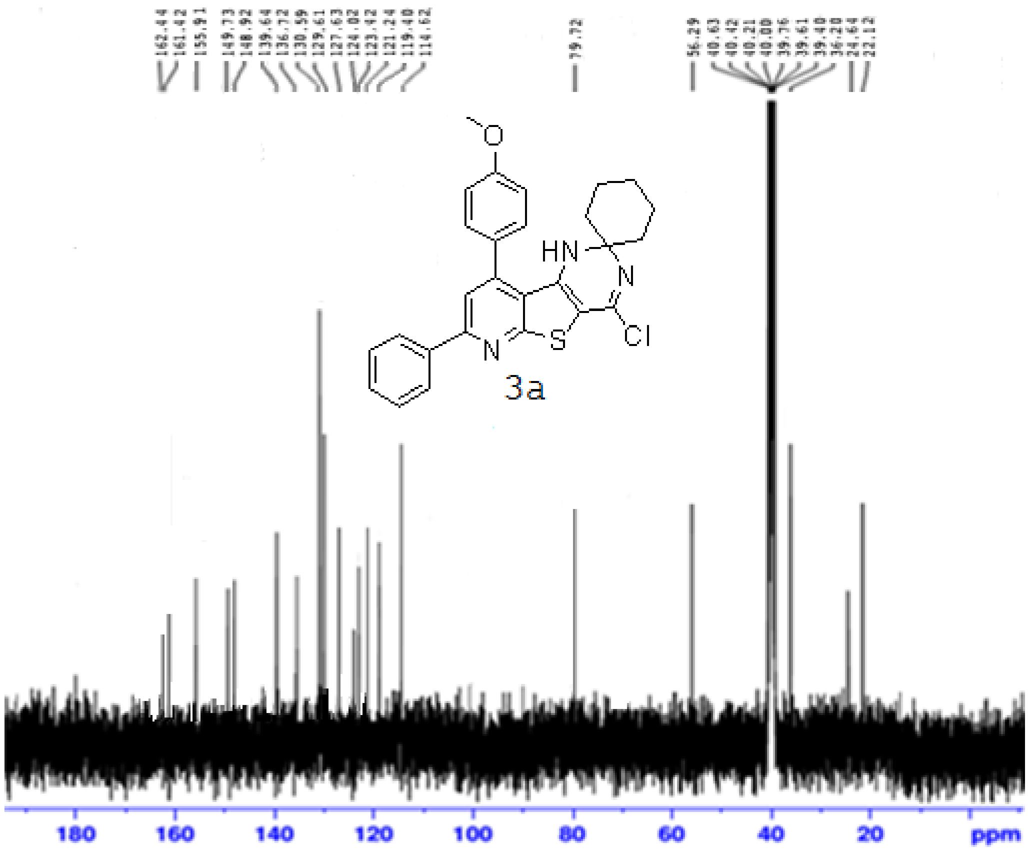

Fig. S6 ^{13}C NMR (100 MHz) in DMSO-d_6 of compound **3a**

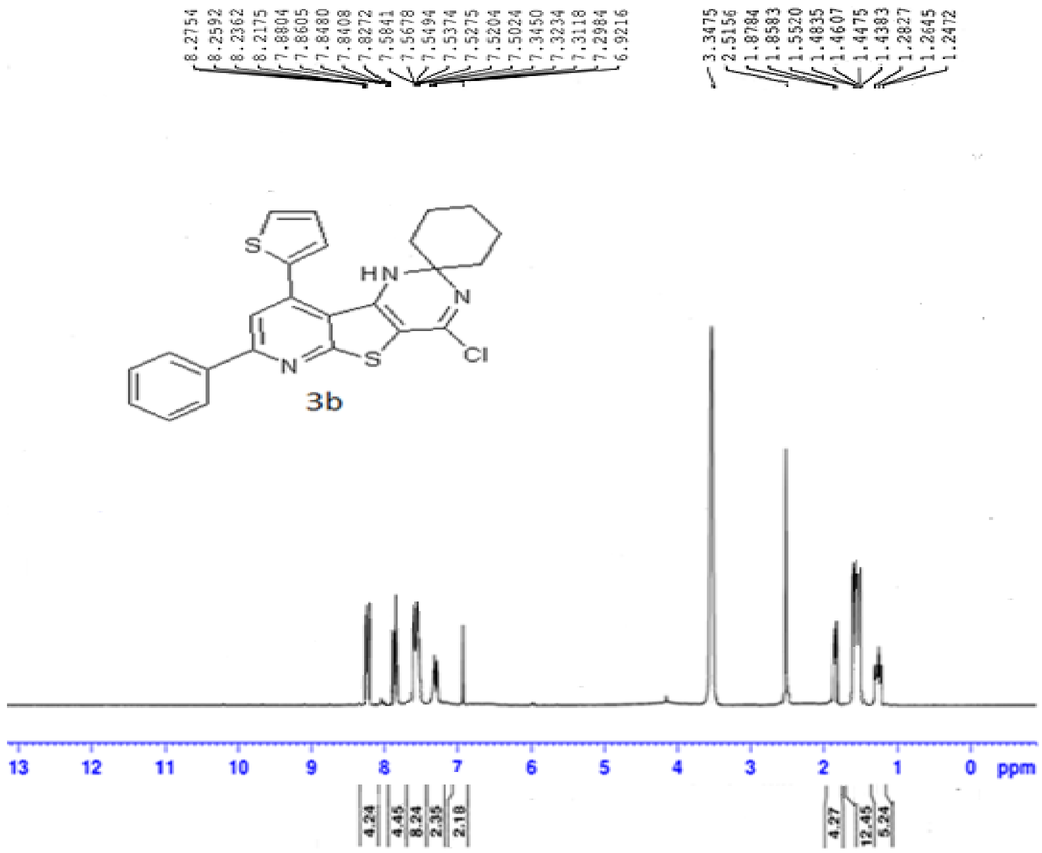

Fig. S7 ^{1}H NMR (400 MHz) in DMSO-d_6 of compound **3b**

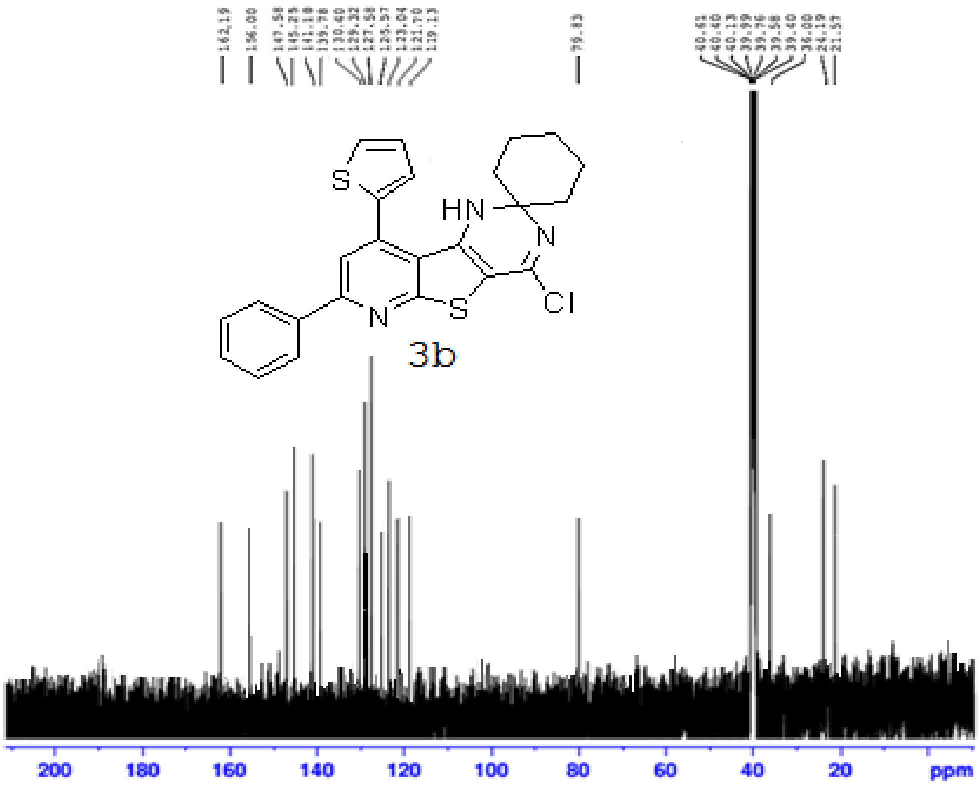

Fig. S8 ^{13}C NMR (100 MHz) in DMSO-d_6 of compound **3b**

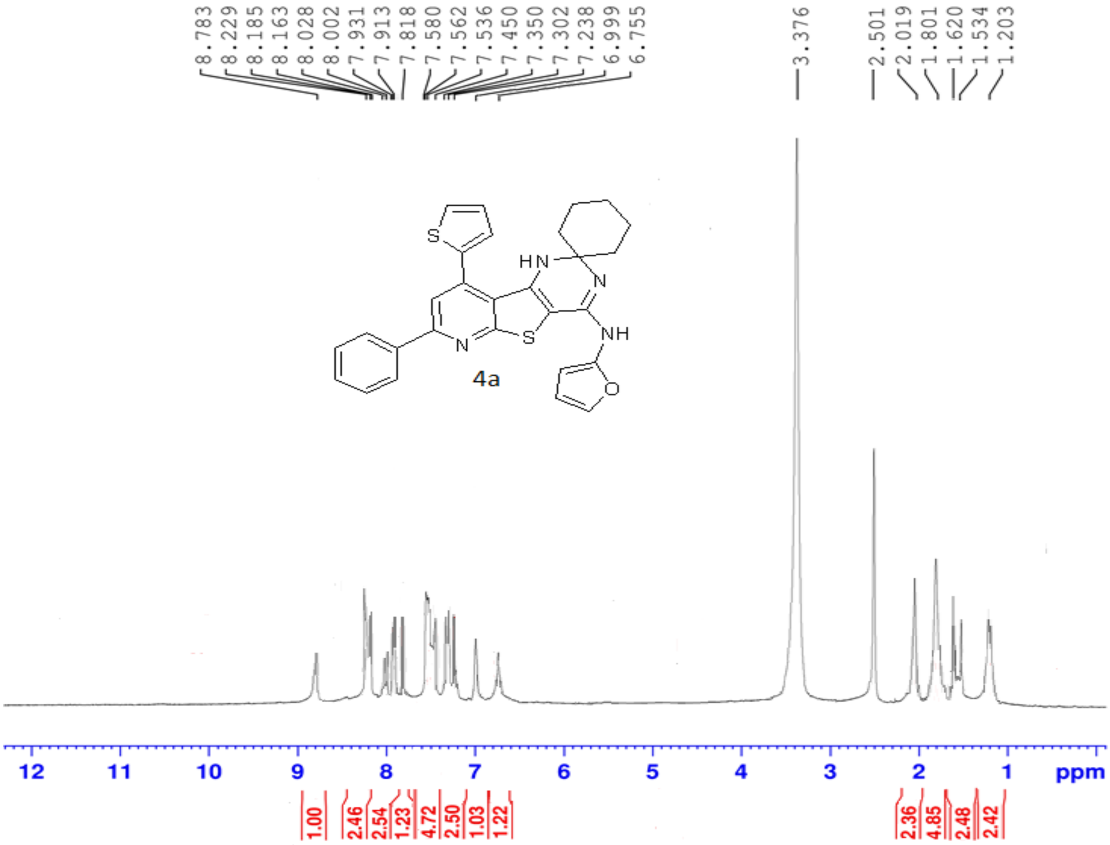

Fig. S9 ^{1}H NMR (400 MHz) in DMSO-d_6 of compound **4a**

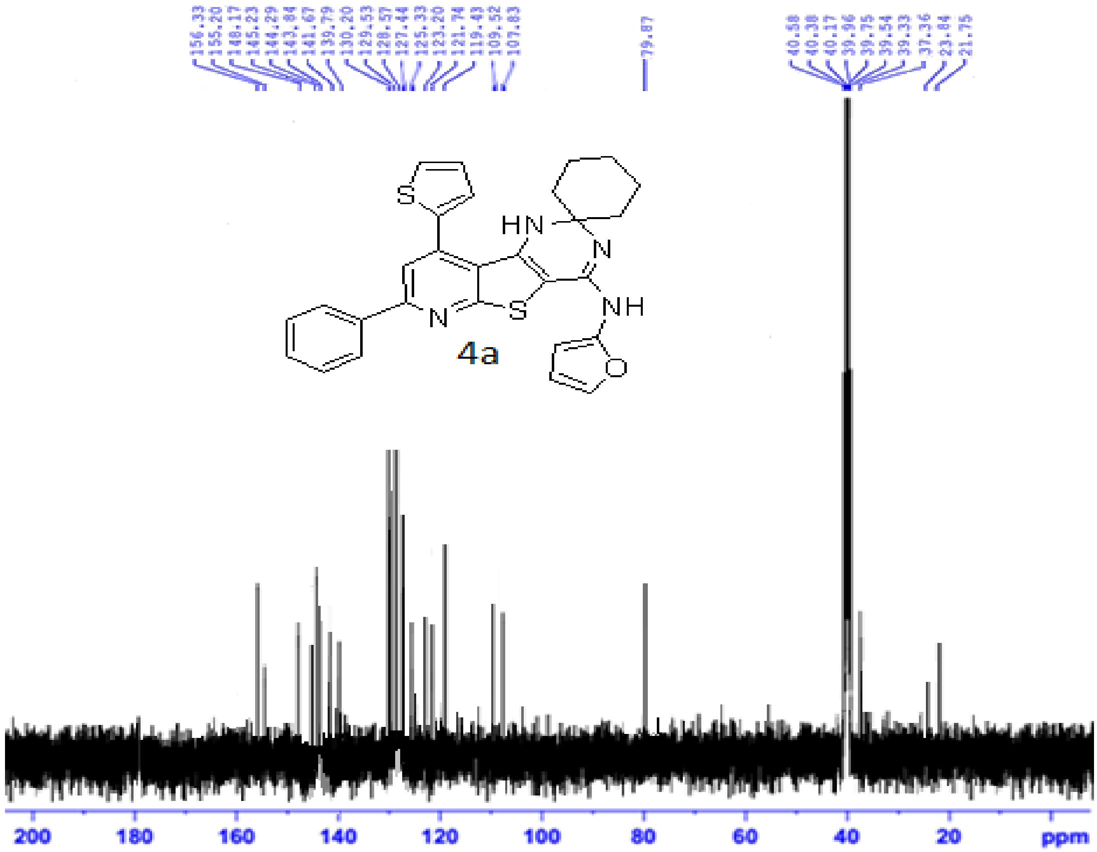

Fig. S10 ^{13}C NMR (100 MHz) in DMSO-d_6 of compound **4a**

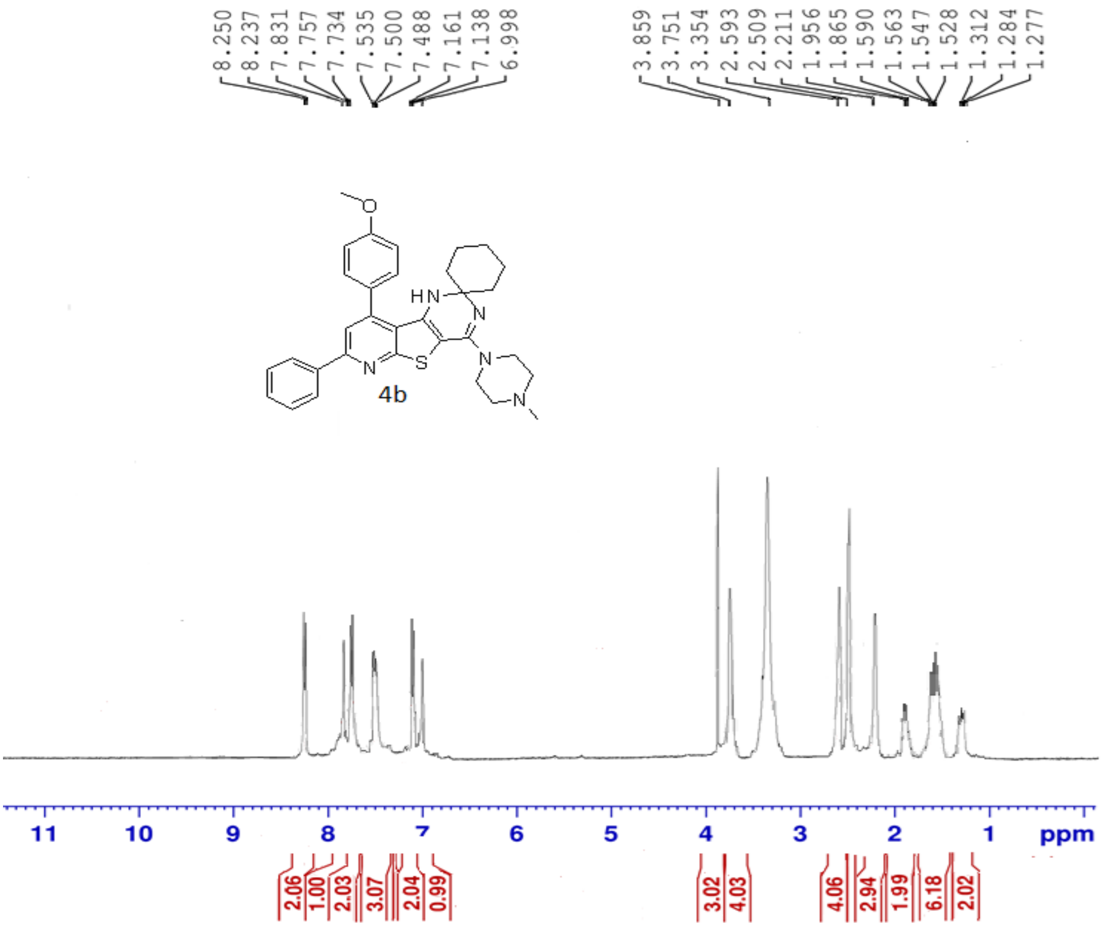

Fig. S11 ^{1}H NMR (400 MHz) in DMSO-d_6 of compound **4b**

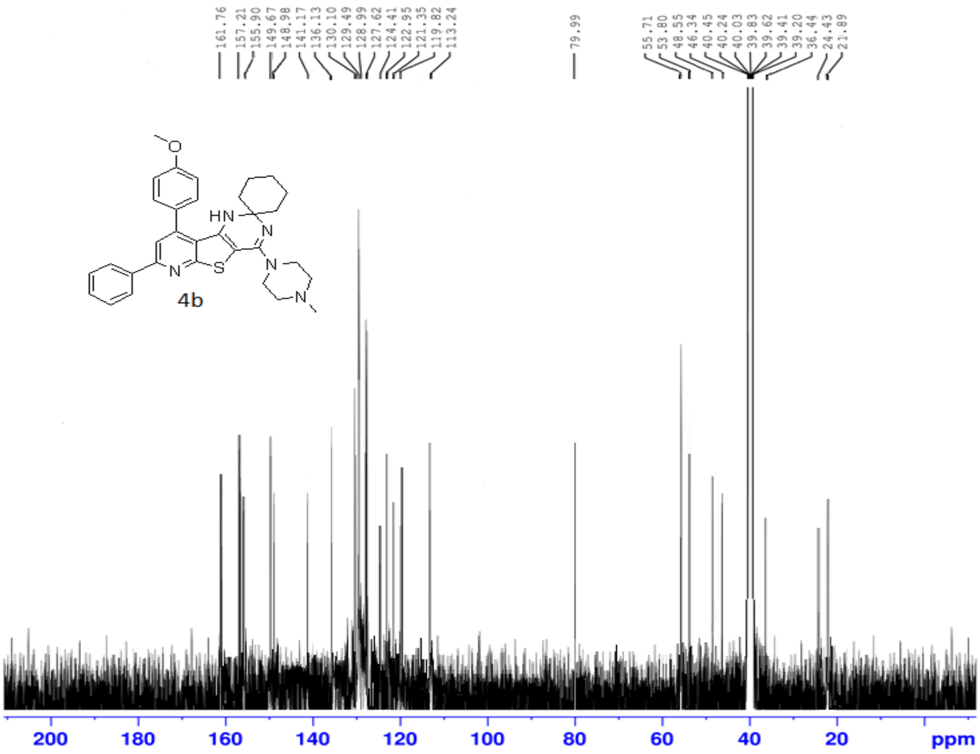

Fig. S12 ^{13}C NMR (100 MHz) in DMSO-d_6 of compound **4b**

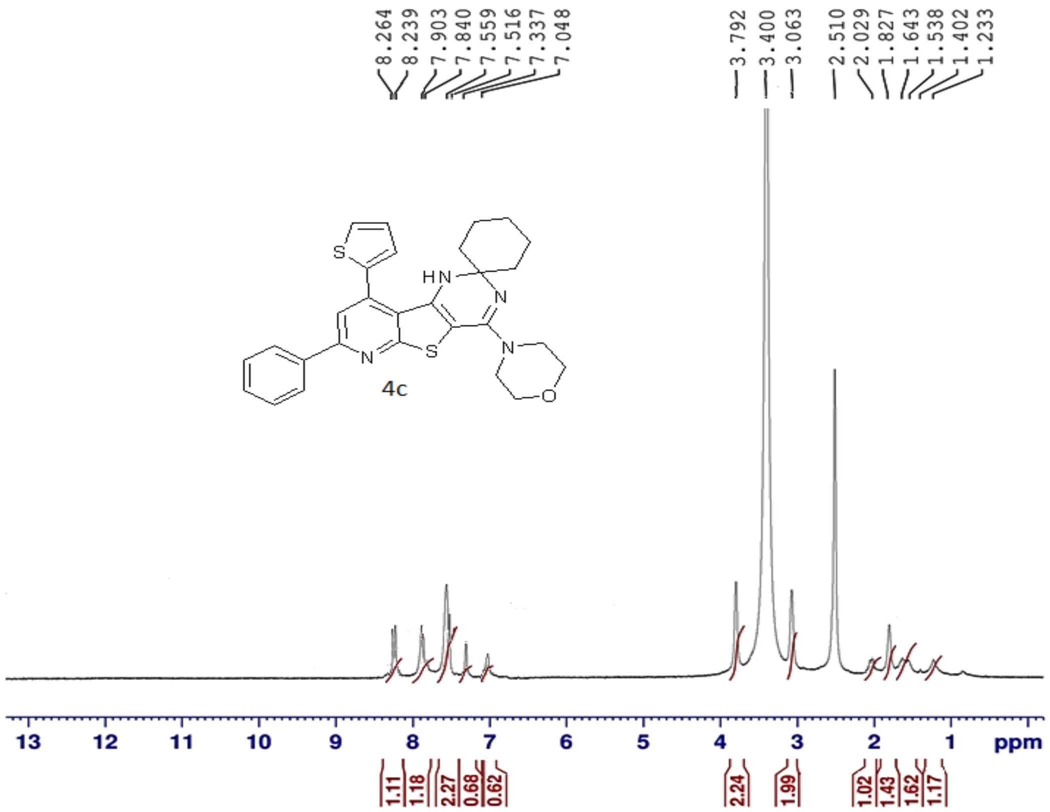

Fig. S13 ^{1}H NMR (400 MHz) in DMSO-d_6 of compound **4c**

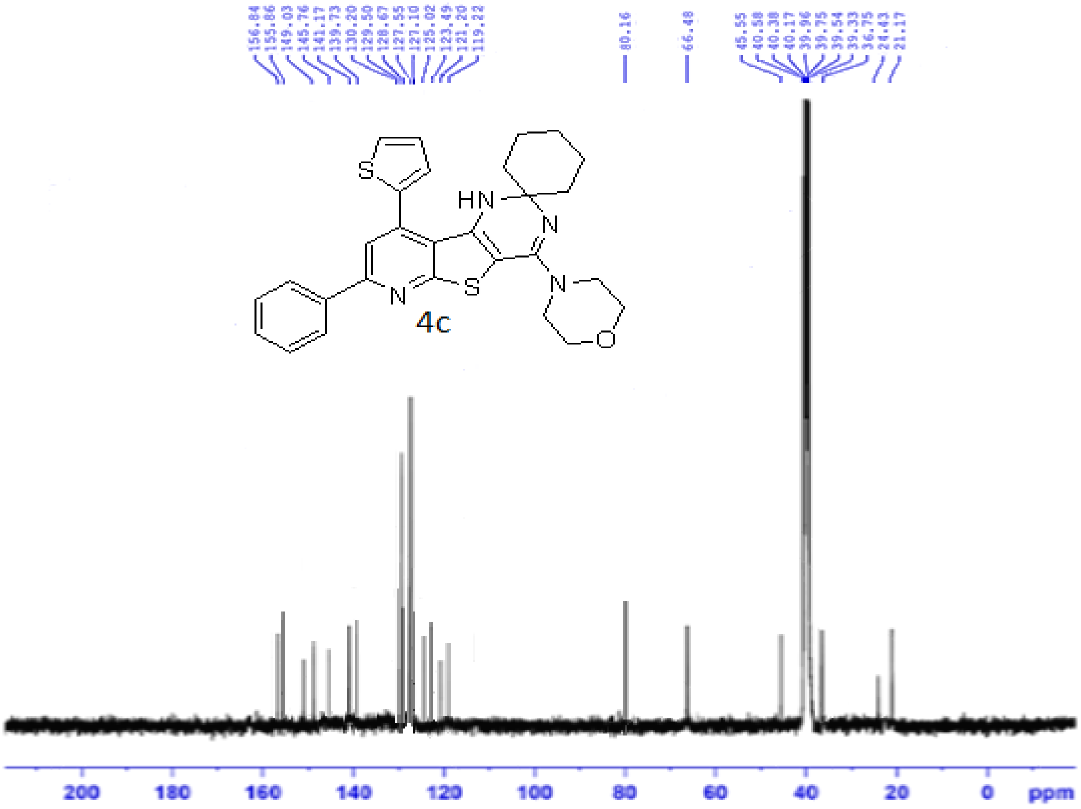

Fig. S14 ^{13}C NMR (100 MHz) in DMSO-d_6 of compound **4c**

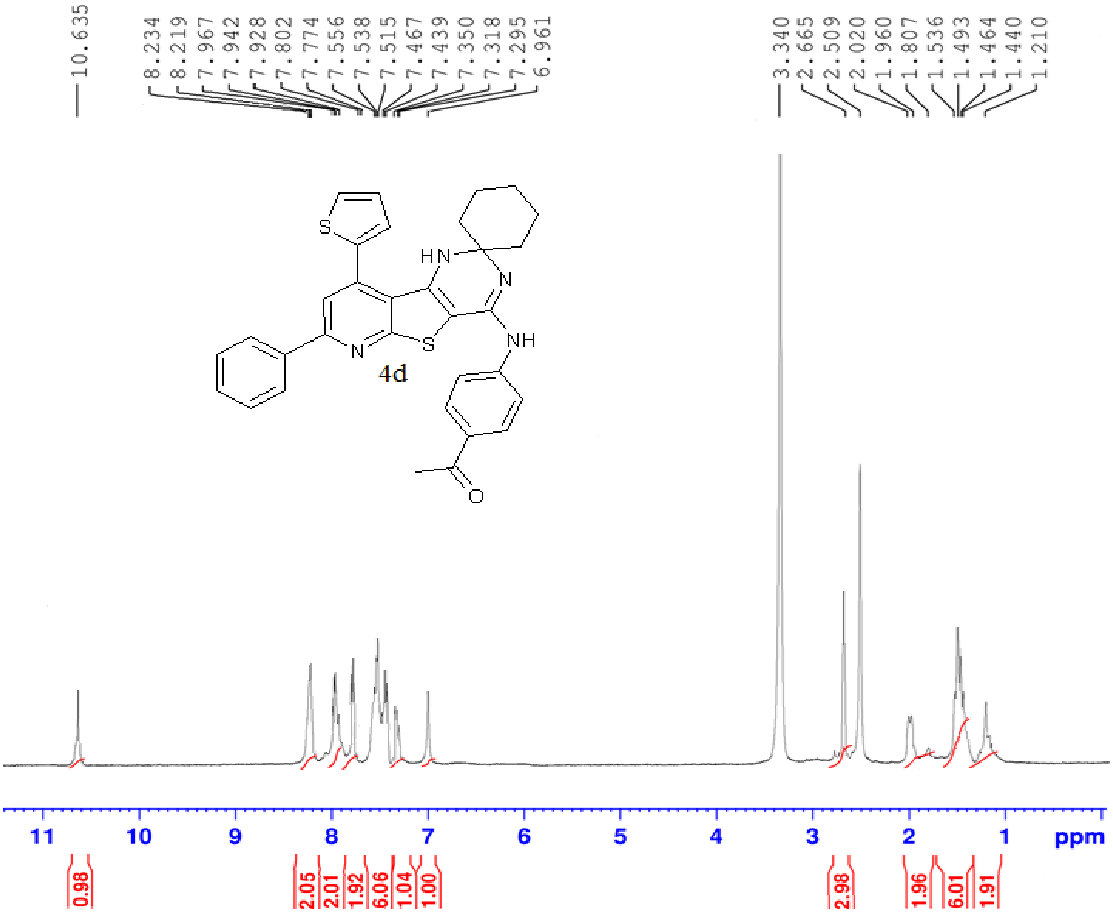

Fig. S15 ^{1}H NMR (400 MHz) in DMSO-d_6 of compound **4d**

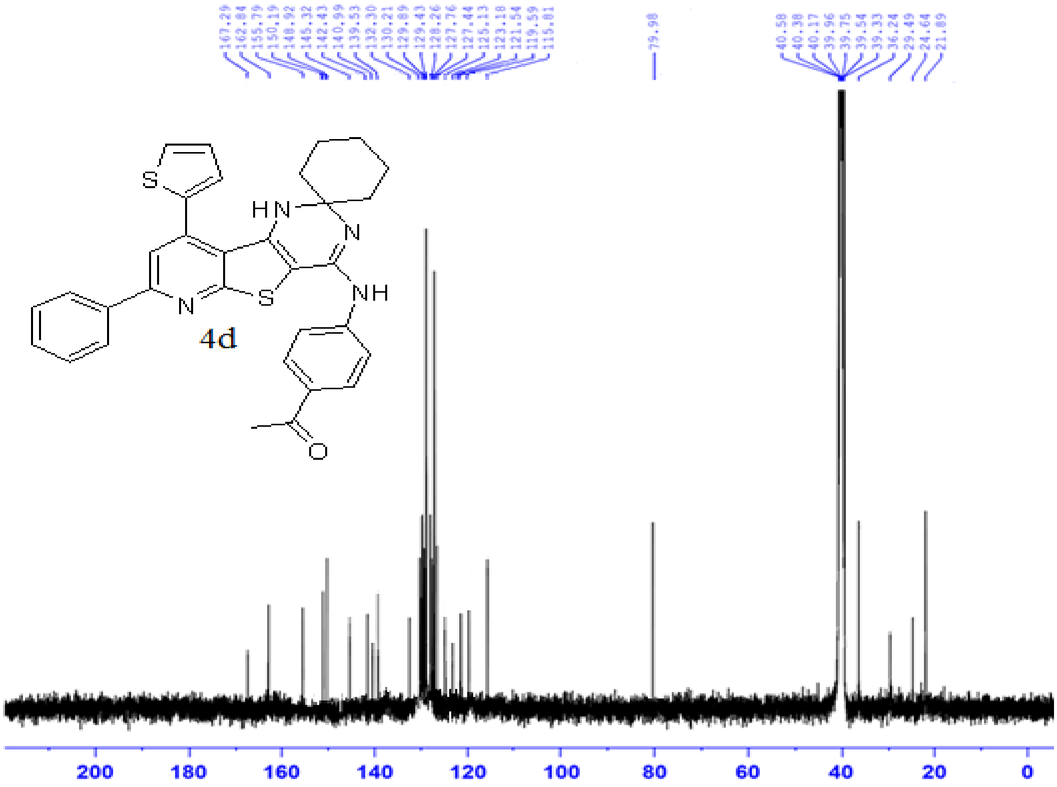

Fig. S16 ^{13}C NMR (100 MHz) in DMSO-d_6 of compound **4d**

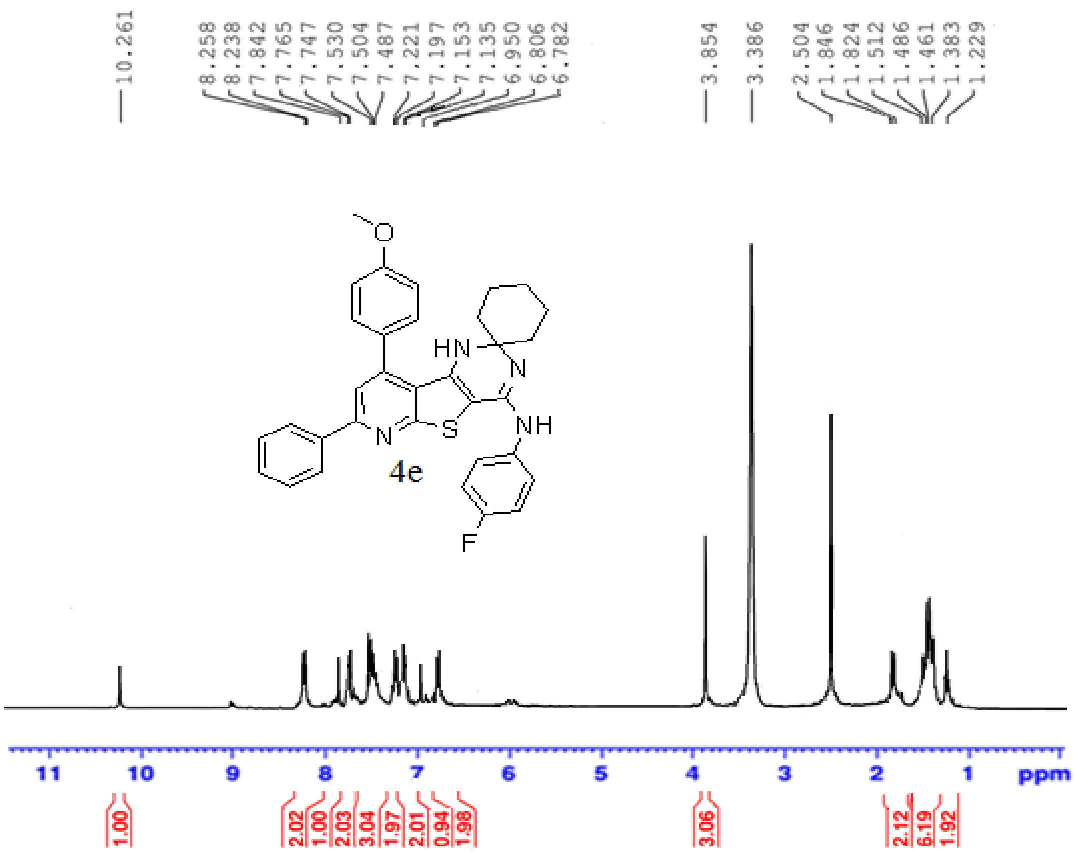

Fig. S17 ^{1}H NMR (400 MHz) in DMSO-d_6 of compound **4e**

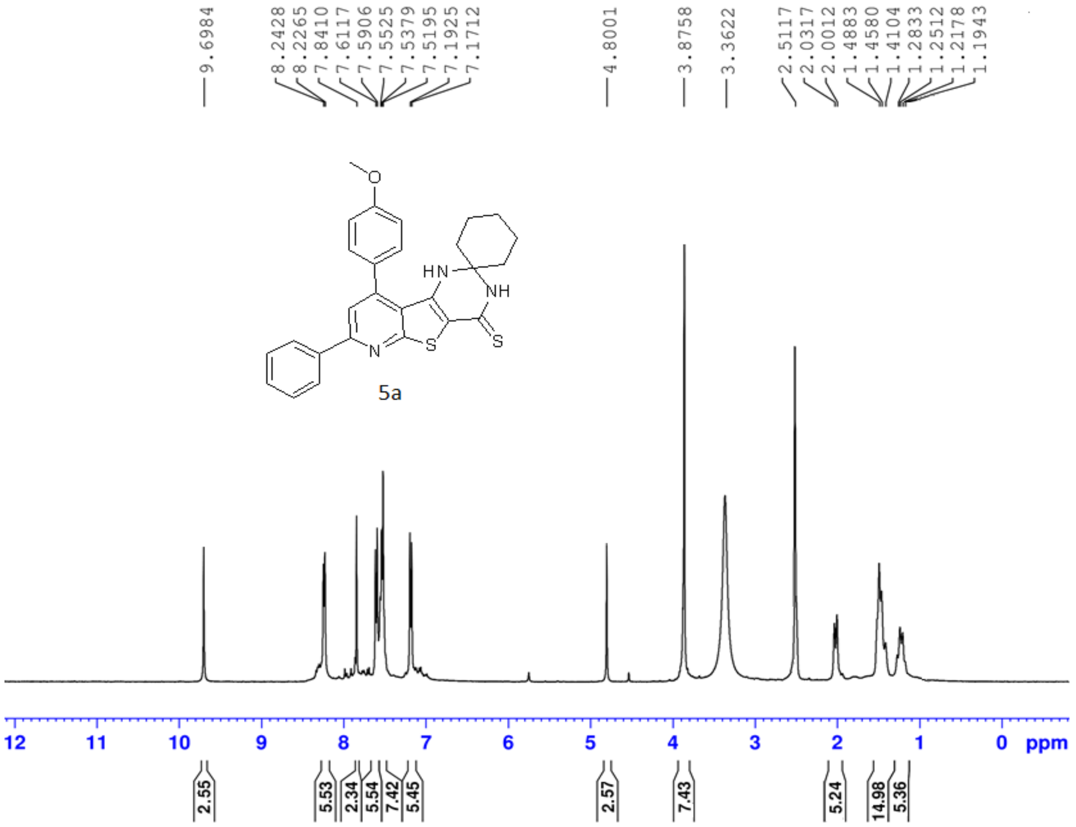

Fig. S18 ^{1}H NMR (400 MHz) in DMSO-d_6 of compound **5a**

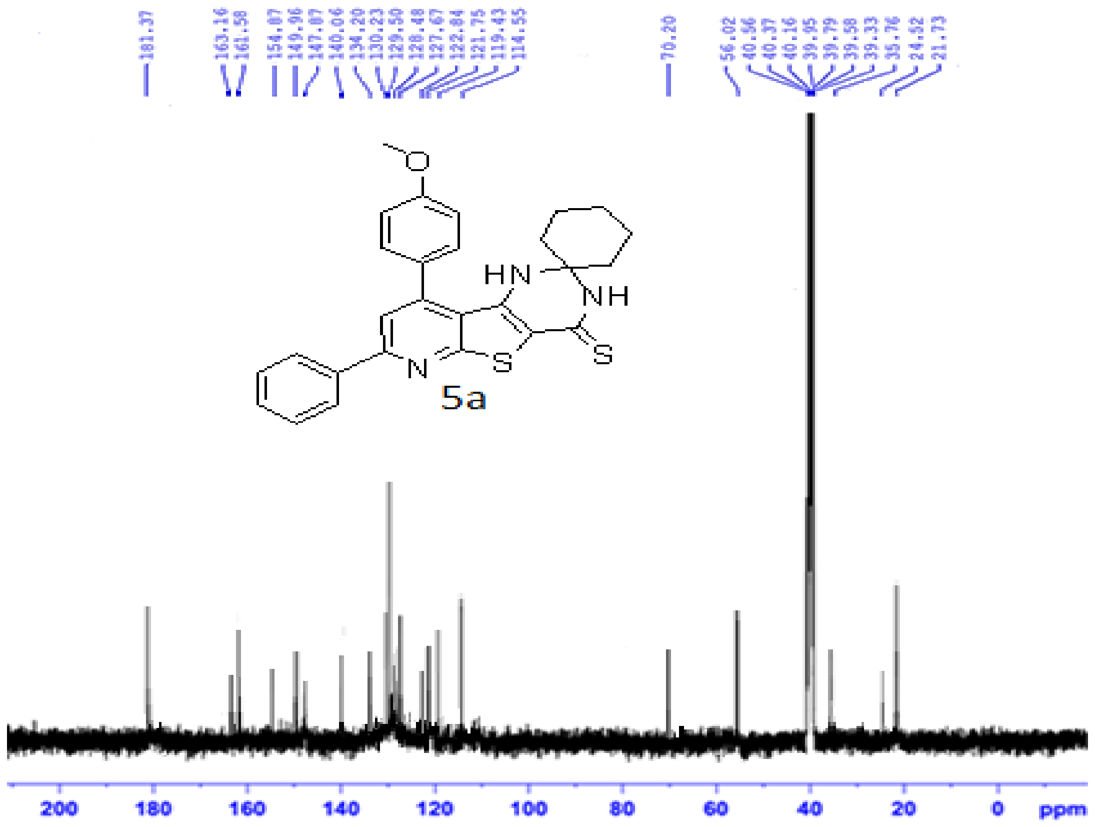

Fig. S19 ^{13}C NMR (100 MHz) in DMSO-d_6 of compound **5a**

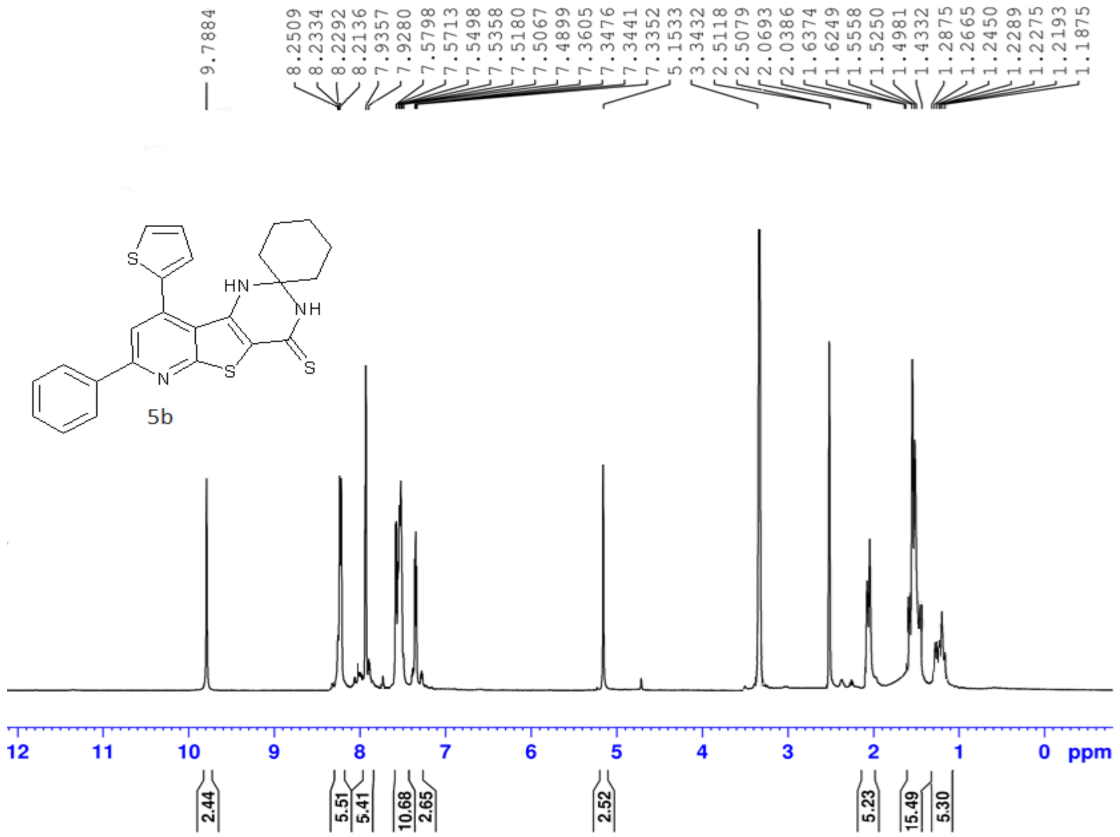

Fig. S20 ^{1}H NMR (400 MHz) in DMSO-d_6 of compound **5b**

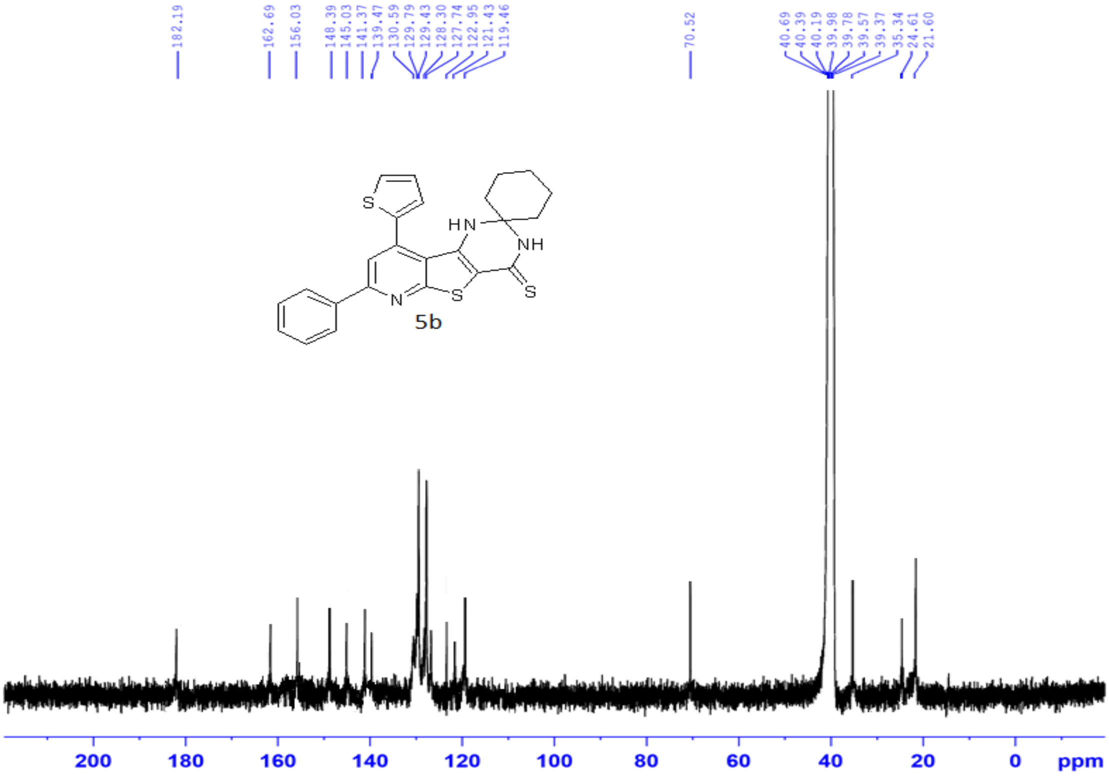

Fig. S21 ^{13}C NMR (100 MHz) in DMSO-d_6 of compound **5b**

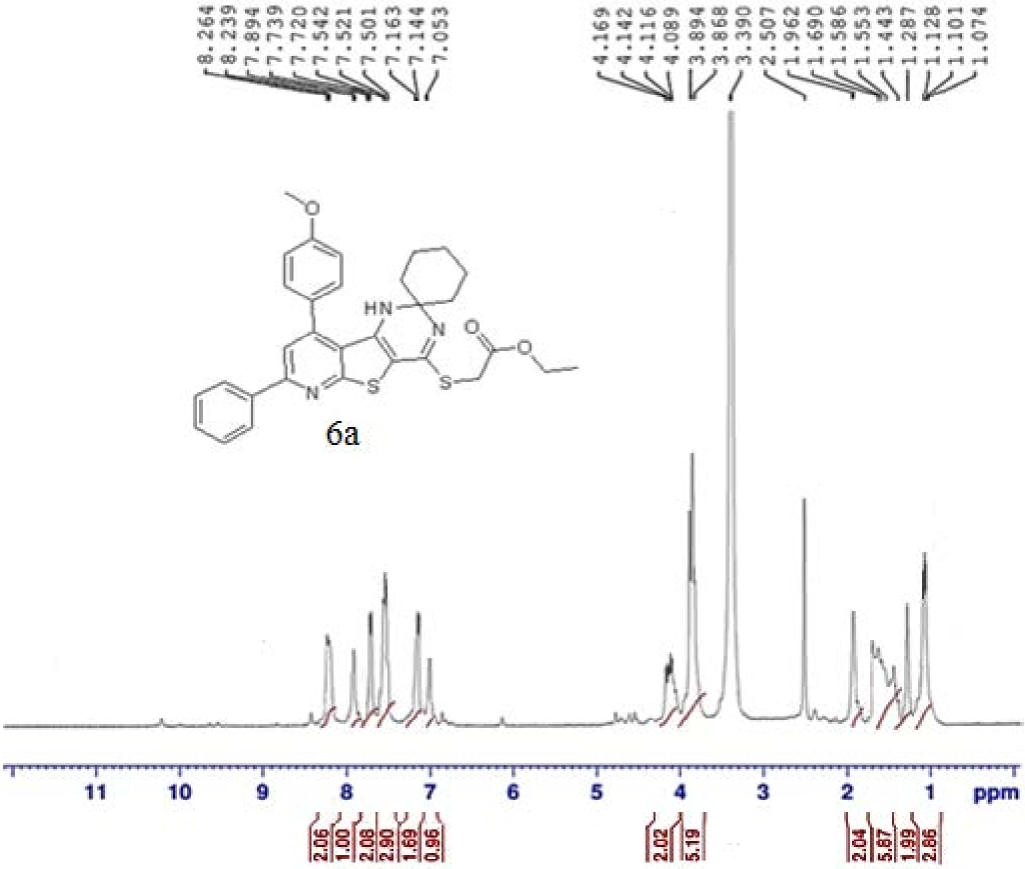

Fig. S22 ^{1}H NMR (400 MHz) in DMSO-d_6 of compound **6a**

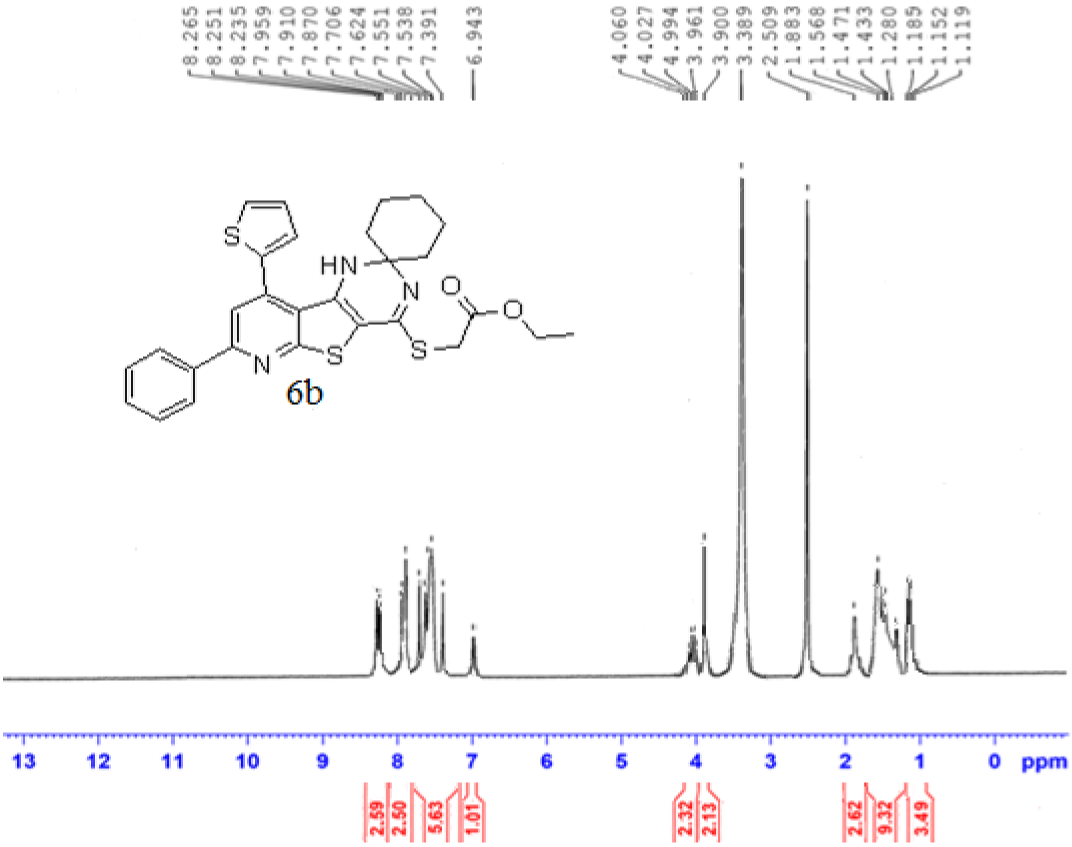

Fig. S23 ^{1}H NMR (400 MHz) in DMSO-d_6 of compound **6b**

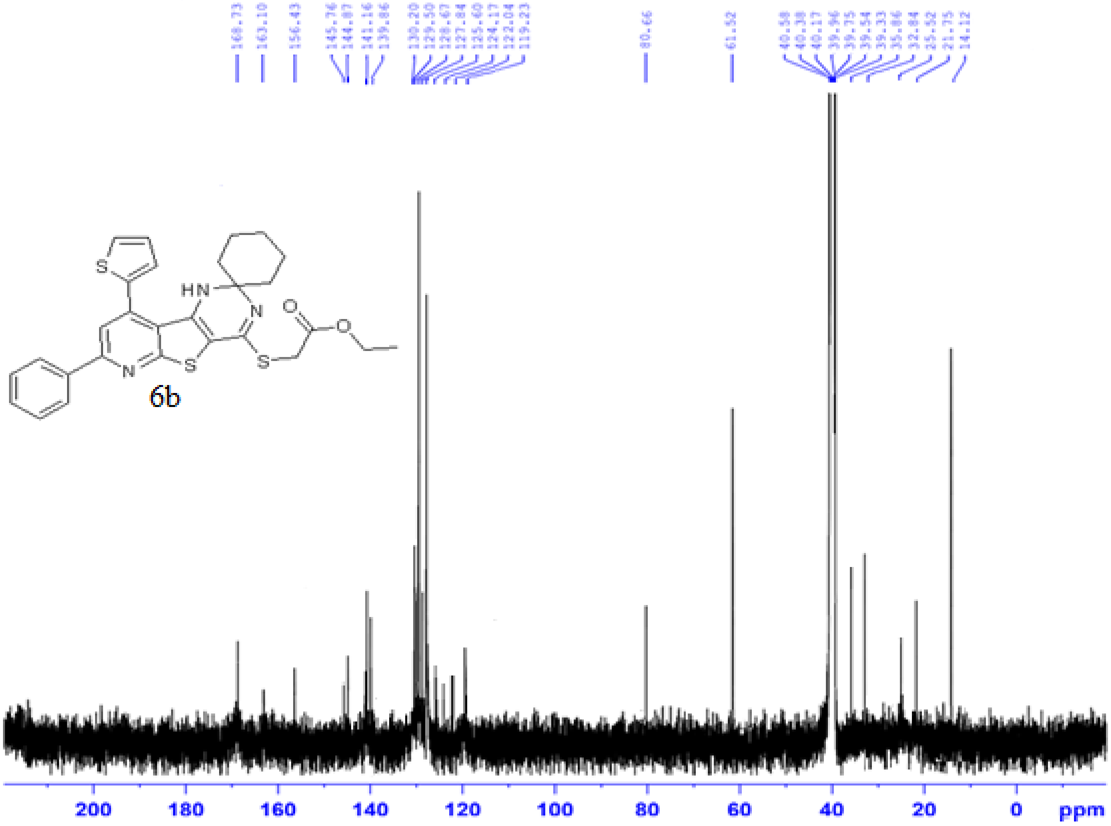

Fig. S24 ^{13}C NMR (100 MHz) in DMSO-d_6 of compound **6b**

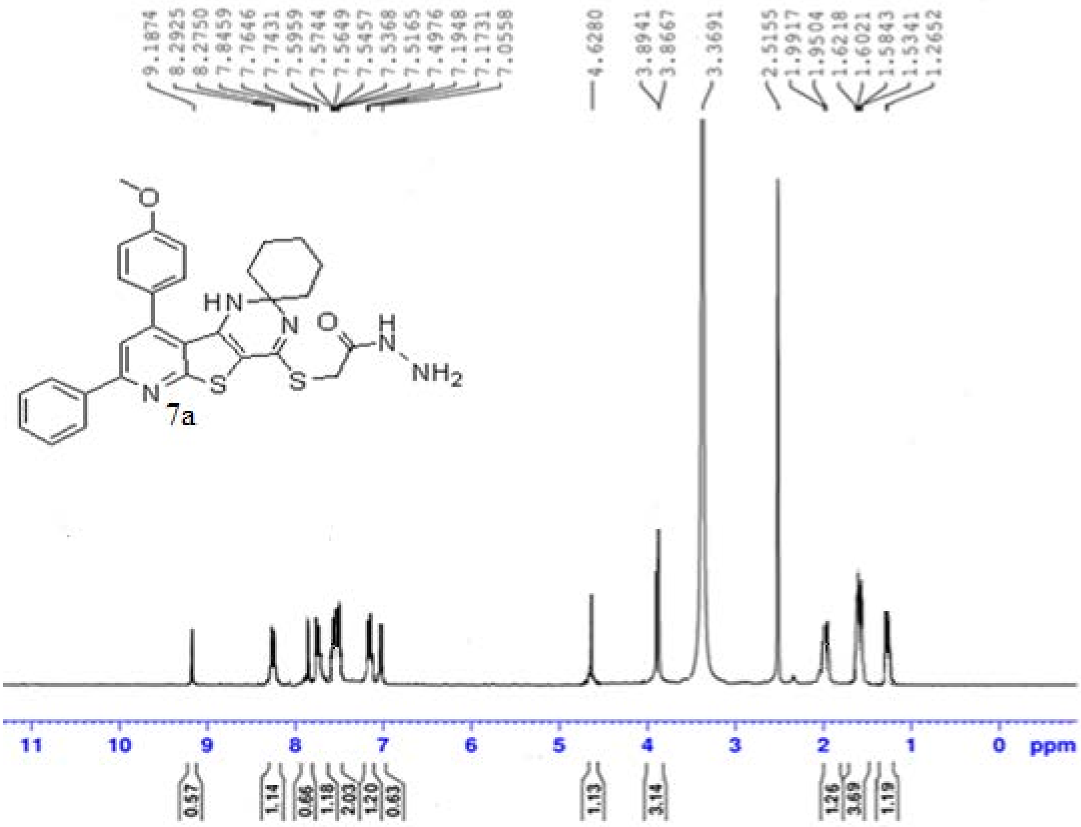

Fig. S25 ^{1}H NMR (400 MHz) in DMSO-d_6 of compound **7a**.

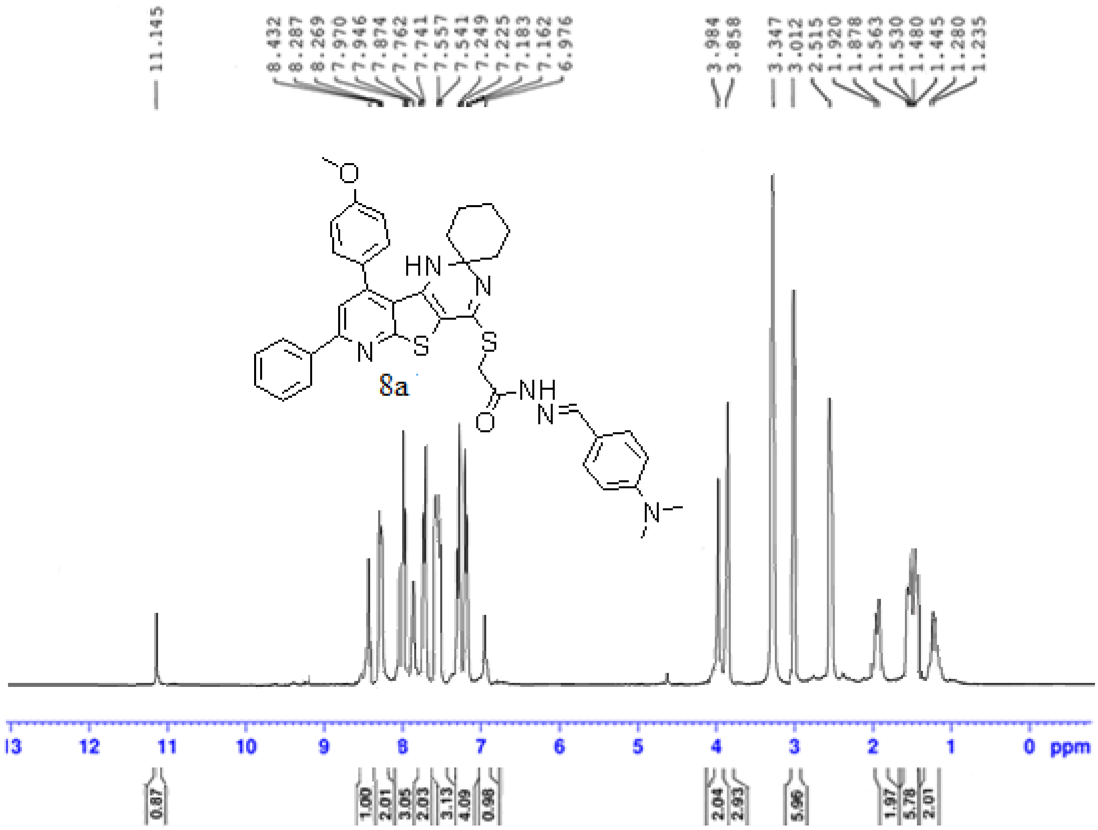

Fig. S26 ^{1}H NMR (400 MHz) in DMSO-d_6 of compound **8a**

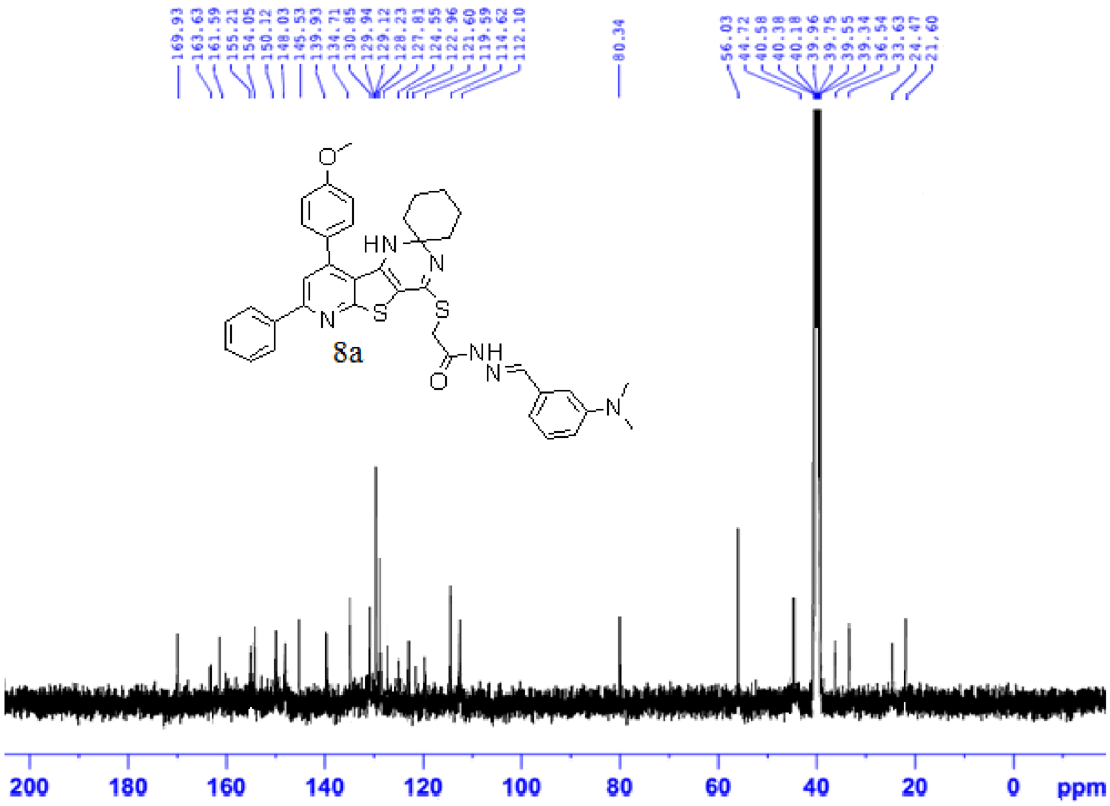

Fig. S27 ^{13}C NMR (100 MHz) in DMSO-d_6 of compound **8a**

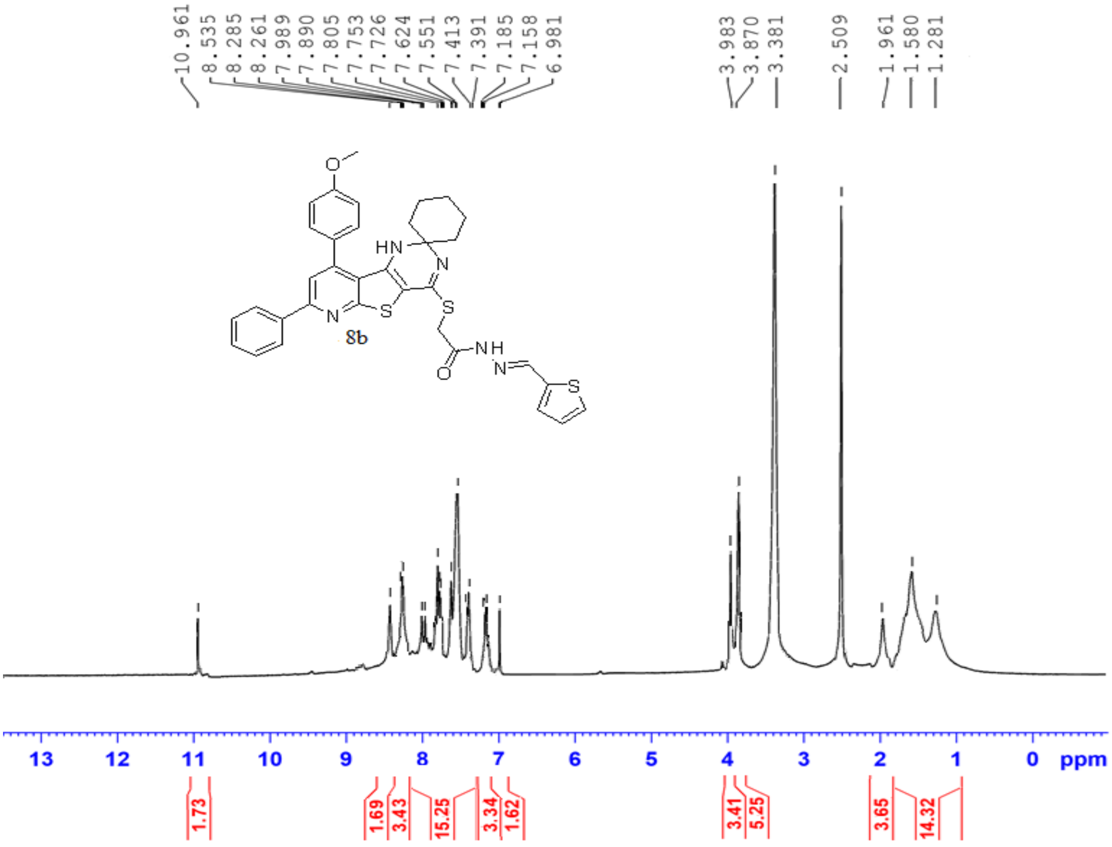

Fig. S28 ^{1}H NMR (400 MHz) in DMSO-d_6 of compound **8b**
